# Amino acid-linked platinum(II) compounds: non-canonical nucleoside preferences and influence on glycosidic bond stabilities

**DOI:** 10.1007/s00775-019-01693-y

**Published:** 2019-07-29

**Authors:** Bett Kimutai, C. C. He, Andrew Roberts, Marcel L. Jones, Xun Bao, Jun Jiang, Zhihua Yang, M. T. Rodgers, Christine S. Chow

**Affiliations:** Department of Chemistry, Wayne State University, Detroit, MI 48202 USA

**Keywords:** Cisplatin, Amino acid-linked platinum(II) compounds, Kinetics, Adenosine adduct, Glycosidic bonds

## Abstract

**Abstract:**

Nucleobases serve as ideal targets where drugs bind and exert their anticancer activities. Cisplatin (cisPt) preferentially coordinates to 2′-deoxyguanosine (dGuo) residues within DNA. The dGuo adducts that are formed alter the DNA structure, contributing to inhibition of function and ultimately cancer cell death. Despite its success as an anticancer drug, cisPt has a number of drawbacks that reduce its efficacy, including repair of adducts and drug resistance. Some approaches to overcome this problem involve development of compounds that coordinate to other purine nucleobases, including those found in RNA. In this work, amino acid-linked platinum(II) (AAPt) compounds of alanine and ornithine (AlaPt and OrnPt, respectively) were studied. Their reactivity preferences for DNA and RNA purine nucleosides (i.e., 2′-deoxyadenosine (dAdo), adenosine (Ado), dGuo, and guanosine (Guo)) were determined. The chosen compounds form predominantly monofunctional adducts by reacting at the N1, N3, or N7 positions of purine nucleobases. In addition, features of AAPt compounds that impact the glycosidic bond stability of Ado residues were explored. The glycosidic bond cleavage is activated differentially for AlaPt-Ado and OrnPt-Ado isomers. Formation of unique adducts at non-canonical residues and subsequent destabilization of the glycosidic bonds are important features that could circumvent platinum-based drug resistance.

**Graphic abstract:**

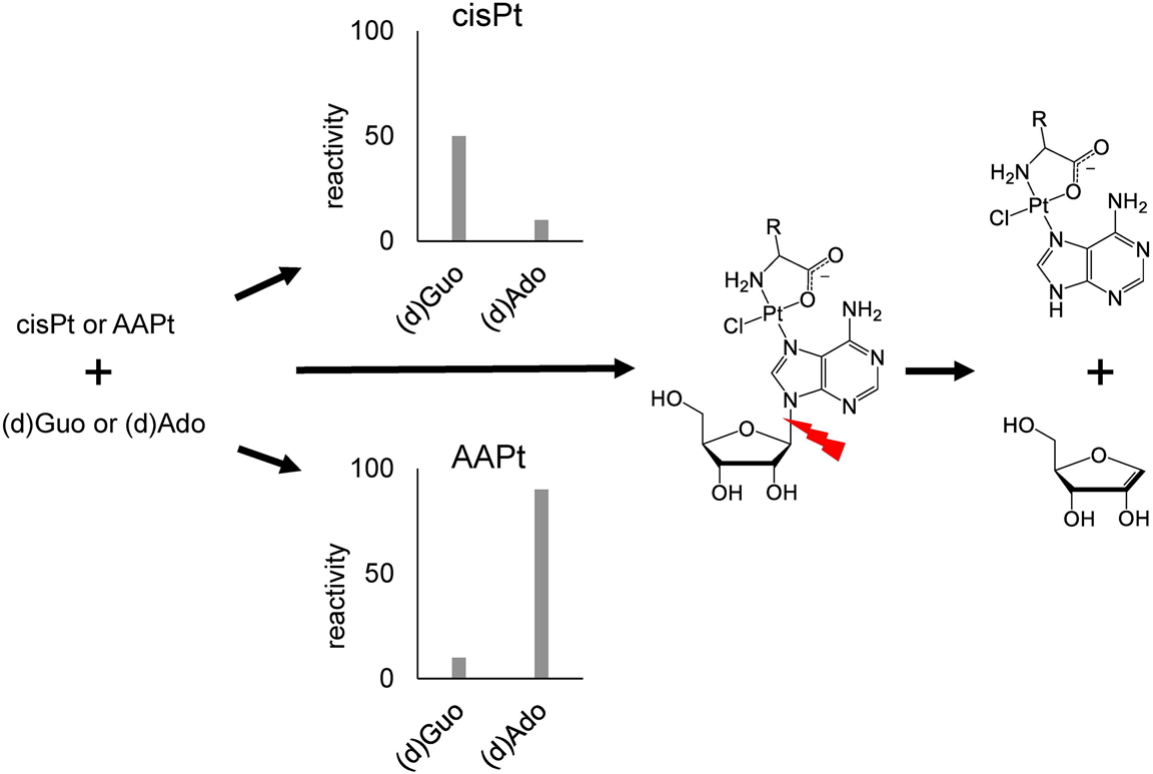

**Electronic supplementary material:**

The online version of this article (10.1007/s00775-019-01693-y) contains supplementary material, which is available to authorized users.

## Introduction

**Fig. 1:**
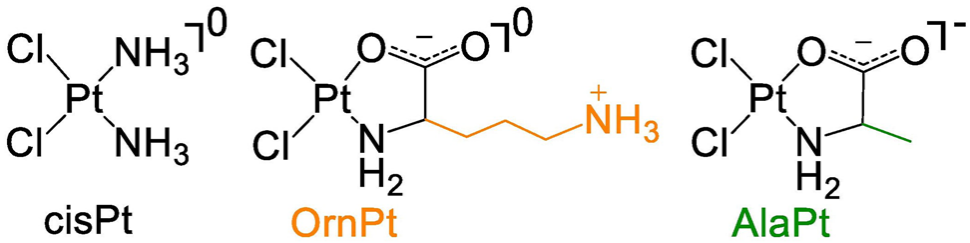
The structures of cisplatin (cisPt) and amino acid-linked platinum(II) (AAPt) compounds, OrnPt and AlaPt, are shown

One of the most commonly used anticancer agents is the platinum-based drug *cis*-diamminedichloridoplatinum(II) (cisplatin or cisPt) (Fig. 1). CisPt is particularly effective against testicular, ovarian, non-small cell lung, head, and neck cancers [1]. The mechanism of action of cisPt has been studied extensively over the past 50 years in an effort to understand its selective clinical efficacy [1, 2]. CisPt, in the aquated form, preferentially coordinates to neighboring 2′-deoxyguanosine (dGuo) residues of DNA at the N7 positions to generate intrastrand adducts as the major products [1, 3, 4]. Formation of these adducts alters the DNA structure and inhibits biological processes such as replication and transcription [5]. The adducts trigger other cellular responses, including cell cycle arrest, DNA repair, and apoptosis [6]. Despite cisPt being used to treat a variety of cancer types, its clinical use may be limited by cellular resistance and adverse side effects such as nephrotoxicity [7–9]. Factors such as DNA repair and reduced accumulation of cisPt allow resistant cancer cells to survive drug treatment [9]. It has also been reported that RNA, including the ribosome, is targeted by cisPt, in which adducts with guanosine (Guo) are formed [10–15]. Analogs of cisPt that exhibit altered mechanisms of action such as targeting RNA or altering RNA levels may be able to circumvent resistance by causing irreparable damage and triggering apoptosis [16–18]. There is also evidence that cisPt interacts and forms adducts with proteins at locations with sulfur-, nitrogen-, and oxygen-containing amino acids, and these interactions play a role in cisPt uptake, side effects, and resistance [19–23]. Chemical and analytical methods allow analysis of these various reaction products and adduct structures of cisPt analogs with their targets, so as to complement their applications in biological and clinical assays.

CisPt analogs can be generated with simple ligand modifications. Even small modifications may alter the reactivity preferences of the analogs toward residues other than dGuo. For example, a cisPt analog with a heteroaromatic ligand, 4,4′-dipyrazolylmethane, shows accumulation in A/T-rich regions of DNA and formation of adducts with 2′-deoxyadenosine (dAdo) [24]. Previously, amino acid-linked platinum (II) (AAPt) compounds (Fig. 1) were shown to bind to RNA, and the ornithine derivative (OrnPt) with a propylamine-containing side chain exhibited a reactivity preference for adenosine (Ado) over Guo residues [25].

**Scheme 1:**
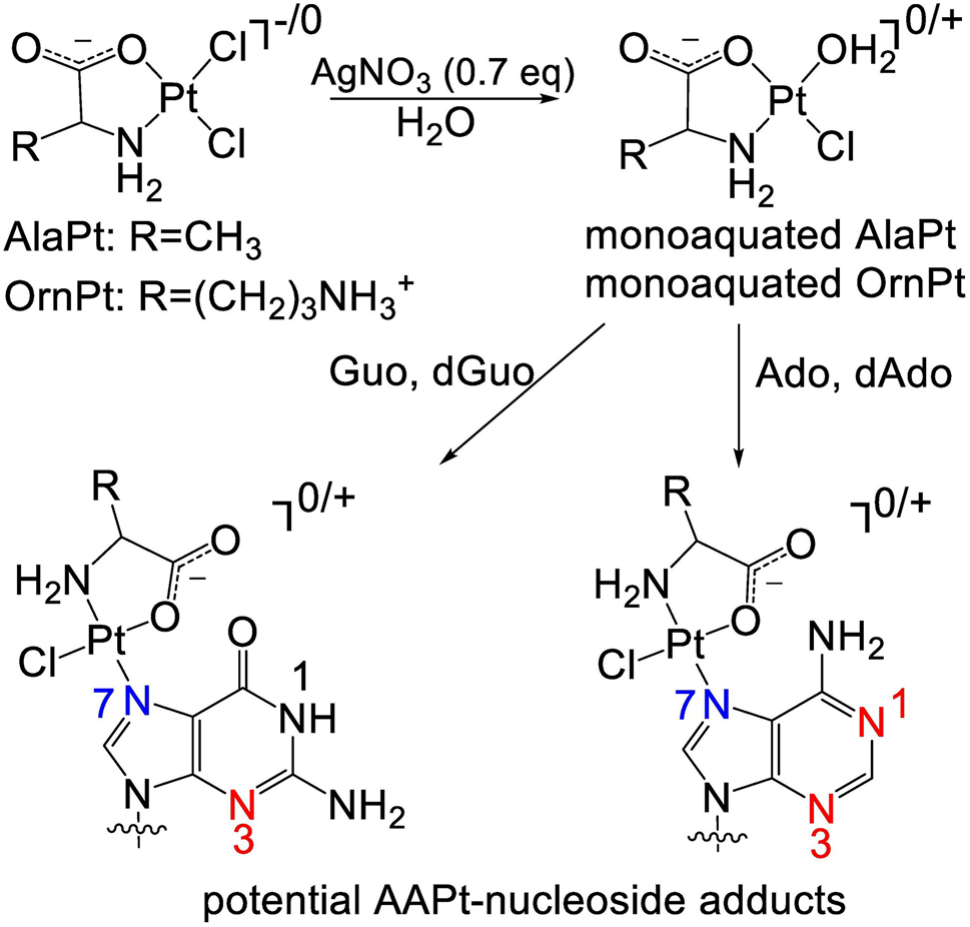
Aquation of platinum compounds and subsequent platination of purine residues. Possible coordination sites are highlighted in blue (N7) and red (N1 and N3)

Platination kinetics have been shown to correlate with cellular activity of cisPt and second-generation analogs [26–29]. For example, *cis*-diammine-1,1-cyclobutane dicarboxylate platinum(II) (carboplatin) is less potent and toxic than cisPt, which is partly attributed to its 100-fold slower adduct formation with DNA [27]. The coordination of platinum-based compounds with DNA involves a number of steps that contribute to the overall reaction rates. Before cisPt reacts with DNA in a cellular setting, it is first aquated to generate *cis*-[PtCl(NH_3_)_2_(H_2_O)]^+^, whereby a chlorido is replaced with H_2_O [30, 31]. Reactions of the aquated cisPt species with DNA then occur under kinetic control, leading to stable adduct formation at the preferred dGuo sites [30, 32]. Therefore, kinetic studies can be used to compare the reactivities of aquated cisPt and AAPt compounds. For in vitro studies, cisPt or AAPt analogs are reacted with one molar equivalent of AgNO_3_ in order to generate the monoaquated species prior to reactions with nucleosides or nucleic acids (Scheme 1) [30]. A number of AAPt-nucleoside adducts form by coordination at the N7 of dGuo/Guo or N1, N3, or N7 positions of dAdo/Ado. By comparing the kinetic rate constants (*k* values) for disappearance of the nucleoside over time, the nucleobase preferences of aquated cisPt and AAPt (i.e., the alanine derivative (AlaPt) and OrnPt, Fig. 1) can be determined [33–35].

The overall process of cisPt adduct formation such as aquation and DNA platination [27, 29] is influenced by a number of factors associated with the non-leaving group ligand (typically an amine), including coordination type and hydrogen-bonding properties [28, 29]. These factors play roles in target preferences and the types of adducts that are formed. CisPt reacts with dGuo of single- and double-stranded DNA leading to the formation of kinetically inert adducts [28, 36, 37]. The adduct forms when the aqua ligand is substituted by a nucleobase atom via a trigonal-bipyramidal transition state [38]. Hydrogen bonding is implicated in the kinetic control of platination [29, 39] and can stabilize the transition state of the aquated cisPt-dGuo reaction [36]. More specifically, computational analysis showed that in the transition state, cisPt forms a strong hydrogen bond between its ammine ligand and the oxo at the 6 position of dGuo [36]. As such, this hydrogen-bonding interaction together with charge–charge interactions results in preferential coordination of cisPt to dGuo [36]. In contrast, formation of the cisPt-Ado adduct is less favored because Ado has an amine at the 6 position, which cannot hydrogen bond with cisPt [36].

In the work presented here, the reaction kinetics of two AAPt compounds were examined to determine how the chemical structures and physical properties of the functional groups of the amino acid (i.e., a short nonpolar side chain vs. a longer more flexible basic side chain) relate to reactivity and nucleoside preference. Such an understanding of reactivity and selectivity is needed to drive the development of new compounds that have improved cellular activities and effectiveness in resistant cells due to the targeting of alternative biological molecules such as RNA [16, 18, 40]. We used pseudo-first-order kinetics to compare the reactivity of the two AAPt compounds. We hypothesized that modification of cisPt with amino acid ligands of varying sizes and charges would alter the reaction kinetics and nucleoside binding preferences. In addition, the type of ligands chosen may also affect the adduct structures (i.e., different nucleoside platination sites such as N1, N3, or N7, and geometric or constitutional isomers, etc.) that are formed, due to unique interactions with the nucleobase functional groups.

Platination of nucleic acids may also have unique influences on the chemical properties of the nucleosides such as bond strengths. For example, modifications such as alkylation or protonation of DNA residues have previously been observed to weaken the glycosidic bond and accelerate the process of depurination [41–44]. Thus, hydrolytic instability of purine glycosidic bonds may impact nucleoside integrity, particularly under varying pH and ionic strength conditions and modification states [42, 45–47]. Stability of the glycosidic bond may also be influenced differentially by platination at varying sites on the nucleobase. In this study, we investigated the impact of AlaPt and OrnPt adducts on relative glycosidic bond strengths by employing energy-resolved collision-induced dissociation (ER-CID) tandem mass spectrometry and survival yield analysis [42, 48–51]. In a previous theoretical study, evaluation of the effects of cisPt modification indicated that platination at N7 does not destabilize the dGuo glycosidic bond [52]. To the best of our knowledge, the influence of platination on the glycosidic bonds of dAdo or Ado adducts has not been investigated, likely because cisPt and other platinum compounds coordinate preferentially with dGuo (or Guo) residues. The integrity of the glycosidic bonds, especially in the Ado residues of RNA, is crucial for cell survival [53]. However, possible destabilization leading to cleavage of the glycosidic bonds in AAPt-Ado adducts could also be exploited as a therapeutic pathway against resistant cancer cells. Because of our observation that some AAPt compounds target Ado residues in RNA [25], we sought to evaluate the relative glycosidic bond strengths of these non-canonical structures. An understanding of AAPt features that modulate glycosidic bond stability is essential for development of such pathways.

## Materials and methods

### Materials

Potassium tetrachloroplatinate(II), l-alanine, trimethylsilyl propionate (TSP), and all nucleosides were purchased from Sigma-Aldrich (St. Louis, MO, USA). The l-ornithine and *cis*-diamminedichloridoplatinum(II) were from Alfa Aesar (Haverhill, MA, USA). Deuterium oxide (D_2_O) was from Cambridge Isotope Laboratories (Tewksbury, MA, USA). Silver nitrate, acetonitrile, methanol, and ammonium acetate were obtained from Fisher Chemical (Hampton, NH, USA). Sodium phosphate, monobasic and dibasic, was obtained from EMD Millipore (Kankakee, IL, USA).

### Synthesis and characterization of AlaPt

Synthesis of AlaPt was carried out following a previously described procedure, but with some modifications to improve yield and minimize undesired side reactions [54]. First, 0.17 mmol (70 mg) of K_2_PtCl_4_ and five equivalents of l-alanine (75 mg) were dissolved in 2 mL of double-distilled water (ddH_2_O) and heated at 50 °C overnight on a Fisher thermoshaker at 950 rpm. The crude product was filtered and vacuum dried for approximately 12 h. Next, 3.0 mL of ice cold ddH_2_O was added to the resulting solid, and the solution was mixed by vortexing for 1 min, followed by centrifugation at 14,000 rpm for 10 min. The supernatant was removed and discarded. To the pellet 3.0 mL of cold 95% ethanol was added, followed by vortexing and centrifugation at 14,000 rpm for 8 min and removal of the supernatant; this procedure was repeated three times. The solid precipitate was then dried under vacuum overnight. To the dried sample 250 µL of 100 mM NaOH was added, followed by vortexing and filtering under centrifugation (Millipore 10 kDa centrifugal filter) at 14,000 rpm for 10 min to remove high molecular weight aggregates. The filtrate was collected and vacuum dried. The final product, obtained in 47% yield (28 mg), was characterized by ^1^H-NMR spectroscopy, ^13^C-NMR, ^195^Pt-NMR, and high-resolution mass spectrometry (Figs. S1, S2, S3, and S4, respectively). Melting point, 249–251 °C; *δ*/ppm (^1^H-NMR, D_2_O, 400 MHz), 3.50 (1 H, q, *J* = 7.1 Hz, =CHCH_3_), 1.28 (3 H, d, *J* = 7.0 Hz, –CH_3_); *δ*/ppm (^13^C-NMR, D_2_O, 400 MHz) 193 ((–COO)CH=), 57 (=CHCH_3_), 20 (=CHCH_3_); *δ*/ppm (^195^Pt-NMR, D_2_O, 500 MHz, − 1639); *m*/*z* (electrospray ionization mass spectrometry (ESI–MS)) 352.943 Da/e [M]^−^; calculated exact mass 352.942 Da [M]^−^; chemical formula PtC_3_H_6_Cl_2_NO_2_ ([M]^−^, at neutral pH).

### Synthesis and characterization of OrnPt

Synthesis of OrnPt was carried out as described previously [54]. First, 0.17 mmol of K_2_PtCl_4_ and two equivalents of l-ornithine (44 mg) were dissolved in 2 mL of ddH_2_O. The mixture was heated at 50 °C overnight on a thermoshaker. The crude product was washed with ice cold ddH_2_O and vacuum dried. The product, obtained in 40% yield (27 mg), was characterized by ^1^H-NMR spectroscopy, high-resolution mass spectrometry, and X-ray crystallography (Figs. S5, S6, and S7). *δ*/ppm (^1^H-NMR, D_2_O, 400 MHz), 3.54 (1 H, t, *J* = 6.3 Hz, –CH(NH_2_)CH_2_–), 2.89 (2 H, t, *J* = 7.9 Hz, –CH_2_NH_3_), 1.84–1.69 (4 H, m, –CH(NH_2_)CH_2_CH_2_–); *m*/*z* (ESI–MS) 395.986 Da/e [M−H]^−^; calculated exact mass 395.984 Da [M−H]^−^; chemical formula PtC_5_H_11_Cl_2_N_2_O_2_ ([M−H]^−^, at neutral pH).

### Aquation of AAPt compounds

Aquation of AlaPt and OrnPt to generate monoaquated AlaPt and monoaquated OrnPt, respectively, involves replacement of one chlorido with an aqua ligand (Scheme 1). The AAPt compound (1.8 µmol) and AgNO_3_ (1.3 µmol, ratio of 1:0.7) were dissolved in 300 µL ddH_2_O and agitated (using a vortex shaker) in the dark at room temperature overnight. The resulting white precipitate (AgCl) was removed through two centrifugation steps at 14,000 rpm for 10 min. The supernatant contained the corresponding aquated AlaPt and OrnPt (i.e., monoaquated AlaPt and monoaquated OrnPt). CisPt was converted to monoaquated cisPt using the same procedure.

### Pseudo-first-order reaction kinetics

The monoaquated AAPt compounds (or monoaquated cisPt) were used in 50-fold excess to produce pseudo-first-order reaction conditions. A 0.1 mM solution of the relevant nucleoside (Ado, Guo, dAdo, dGuo) was incubated with 5 mM of the aquated platinum complex in 25 mM Na_2_HPO_4_/NaH_2_PO_4_ buffer (pH 7) at 37 °C. Six aliquots were collected from the sample at different time points (0, 0.5, 1, 2, 3, 4, and 6 h). Each aliquot was quenched with NaCl (0.12 M final concentration) and then frozen at − 80 °C until HPLC analysis. The HPLC was fitted with a Supelco Discovery C18 column (5 μm particle diameter; 4.6 mm × 250 mm, Sigma-Aldrich, St. Louis, MO, USA) on a Waters 600 LC with a 717plus autosampler (Waters, Milford, MA, USA) and UV detector set at 254 nm. Buffers A (40 mM NH_4_OAc, pH 6.5) and B (40% acetonitrile, 60% ddH_2_O) were employed using isocratic conditions with 5% B for 5 min followed by a linear gradient in which B was increased to 35% over 25 min. The peak area of unreacted nucleoside was monitored over the six time points and its diminishing ratio to the product peaks was determined [55]. The unreacted nucleoside was monitored because its peak was dominant in area and could also be distinguished from other multiple product peaks based on the retention time of the control. The data were fit using an exponential equation assuming pseudo-first-order kinetics (Eq. S1).

### Mass analysis of HPLC fractions

The HPLC fractions containing the AlaPt-Ado and OrnPt-Ado adducts were collected and dried. The samples were dissolved in 50:50 MeOH:ddH_2_O (v/v) and ions were generated with an ESI source. The MS analyses were performed using a 7T Fourier transform ion cyclotron mass spectrometer (FT-ICR MS, solariX, Bruker Daltonics, Billerica, MA, USA). The major ions were identified for each fraction. The dominant AAPt-nucleoside adduct in each fraction was isolated and subjected to CID tandem mass spectrometry analysis. Fragments were observed and assigned based on their measured *m*/*z* values.

### NMR spectroscopy of AAPt-nucleoside adducts

The ^1^H- and ^13^C-NMR (700 MHz) spectra of the AAPt-nucleoside adducts were recorded on a Bruker Avance 700 MHz spectrometer equipped with a TXI cryoprobe (Wayne State Lumigen Instrument Center). The H2 and H8 signals in Ado were assigned by identifying the C2 and C8 chemical shifts in ^13^C-NMR [56] followed by two-dimensional heteronuclear single quantum coherence (HSQC) spectroscopy to correlate C2–H2 and C8–H8. To each sample, 10 μM trimethylsilyl propionate (TSP) was added as an internal standard. The ^1^H chemical shifts were referenced to the residual HDO (semi-heavy water) signal, which was calibrated against TSP. D_2_O (with no buffer) was also chosen as the solvent for the ^1^H- and ^13^C-NMR (700 MHz) spectra of the adducts.

### Energy-resolved collision-induced dissociation (ER-CID) experiments and survival yield analyses

ER-CID experiments of the HPLC fractions containing the AlaPt-Ado and OrnPt-Ado adducts were performed on a quadrupole ion trap mass spectrometer (QIT MS, amaZon ETD, Bruker Daltonics, Billerica, MA, USA). The method has been described in detail in previous work [42, 50, 51]. Briefly, HPLC fractions were vacuum dried and then redissolved in an MeOH:ddH_2_O (50:50 v/v) mixture. Each HPLC fraction (10 μM) was introduced to the ESI source to generate ions, which were then guided into the ion trap. In the trap, ions were accumulated and mass selected for tandem ER-CID experiments. Helium (1 mtorr) served as both the buffer and collision gas to provide efficient trapping and cooling of the ions as well as to achieve fragmentation of the precursor AAPt-nucleoside complex. The q_z_ value was set to 0.25 for the ER-CID experiments, which leads to low mass cutoff of 27% of the precursor ion *m*/*z*. The rf excitation amplitude was increased from 0 V to the rf excitation amplitude required to produce complete dissociation of the precursor ion at a stepsize of 0.01 V. Experiments were performed in triplicate to assess reproducibility. All data were processed using Data Analysis 4.0 (Bruker Daltonics).

The glycosidic bond cleavage (GBC) survival yield was calculated from the intensities of the precursor and fragment ions, and the ratio of the precursor ion intensity to the total ion intensity was determined using Eq. (1) [57]

1 $${\text{Survival}}\;{\text{yield}}\; ( {\text{GBC)}} = {{\left( {\mathop \sum \limits_{x}^{n - x} I_{{{\text{f}}_{i} }} + I_{\text{p}} } \right)} \mathord{\left/ {\vphantom {{\left( {\mathop \sum \limits_{x}^{n - x} I_{{{\text{f}}_{i} }} + I_{\text{p}} } \right)} {\left( {\mathop \sum \limits_{i} I_{{{\text{f}}_{i} }} + I_{\text{p}} } \right)}}} \right. \kern-0pt} {\left( {\mathop \sum \limits_{i} I_{{{\text{f}}_{i} }} + I_{\text{p}} } \right)}}$$

in which $I_{{{\text{f}}_{i} }}$ and $I_{\text{p}}$ are the ion intensities of the fragment and precursor ions, respectively; *x* represents fragments in which the glycosidic bond remains intact. Data analyses were performed using SigmaPlot 10.0 (Systat Software, Inc., San Jose, CA, USA) and custom software developed in the Rodgers laboratory and reported previously [42, 48–51]. The GBC survival yield was plotted as a function of the rf excitation amplitude. The rf excitation amplitude required to produce 50% glycosidic bond cleavage (GBC_50%_) was extracted by fitting of the survival yield curve using the four-parameter logistic dynamic equation (Eq. 2) [42, 50, 51]:

2 $${\text{Survival}}\;{\text{yield}}\;({\text{GBC}}_{50\% } ) = \hbox{min} + (\hbox{max} - \hbox{min} )/\left[ {1 + ({\text{rf}}_{\text{EA}} /{\text{GBC}}_{50\% } } \right]^{{{\text{GBC}}\;{\text{slope}}}}$$

In this equation, max and min are defined as the maximum (1) and minimum (0) values of the survival yield, rf_EA_ is the rf excitation amplitude applied to induce fragmentation, and GBCslope is the slope of the declining region of the survival yield curve.

## Results and discussion

### Synthesis and characterization of amino acid-linked platinum(II) compounds

The synthesized AlaPt and OrnPt were characterized using ^1^H-, ^13^C-, and ^195^Pt-NMR spectroscopy (Figs. S1, S2, S3, and S5) and mass spectrometry (Figs. S4 and S6). In the ^1^H-NMR spectrum, the H_α_ proton peak for AlaPt is shifted upfield by 0.11 ppm compared to the corresponding peak of l-alanine, consistent with coordination of the amino acid to the platinum center. The chemical shift change for the H_β_ proton is 0.03 ppm. In the ^13^C-NMR spectrum, the carboxylate carbon peak for AlaPt is shifted by 17 ppm compared to the corresponding peak of l-alanine (Fig. S2). Characterization of AlaPt by ^195^Pt-NMR shows a shift in the ^195^Pt peak from − 1607 ppm for the potassium tetrachloroplatinate(II) starting material to − 1639 ppm for the AlaPt product (Fig. S3). The mass data are consistent with one amino acid and two chlorido ligands, with AlaPt having (N,O) type of binding (platinum coordination to the N and O atoms of alanine) (Fig. S4). Similarly, the H_α_ proton peak for OrnPt is shifted upfield by 0.09 ppm compared to the corresponding peak of l-ornithine, consistent with metal coordination. The mass data for OrnPt (Fig. S6) confirm coordination of one amino acid to the metal center, but do not reveal (N,O) vs. (N,N) coordination. In this case, the propylamine side chain of ornithine could also coordinate to the metal center. The structure of OrnPt was, therefore, analyzed using X-ray crystallography (Fig. S7), which shows that the platinum maintains a square planar geometry and is part of a five-membered ring with (N,O) coordination, consistent with previous reports [54]. The crystallization conditions for OrnPt may favor the (N,O) type, but the presence of (N,N) type structures in solution cannot be ruled out [58]. The leaving (chlorido) and non-leaving ligands (amino acids), including (N,O) and (N,N) binding, of AAPt were expected to dictate the reactivity of the compounds uniquely compared to cisPt.

### Kinetics of platination reactions with DNA/RNA purine nucleosides

**Fig. 2:**
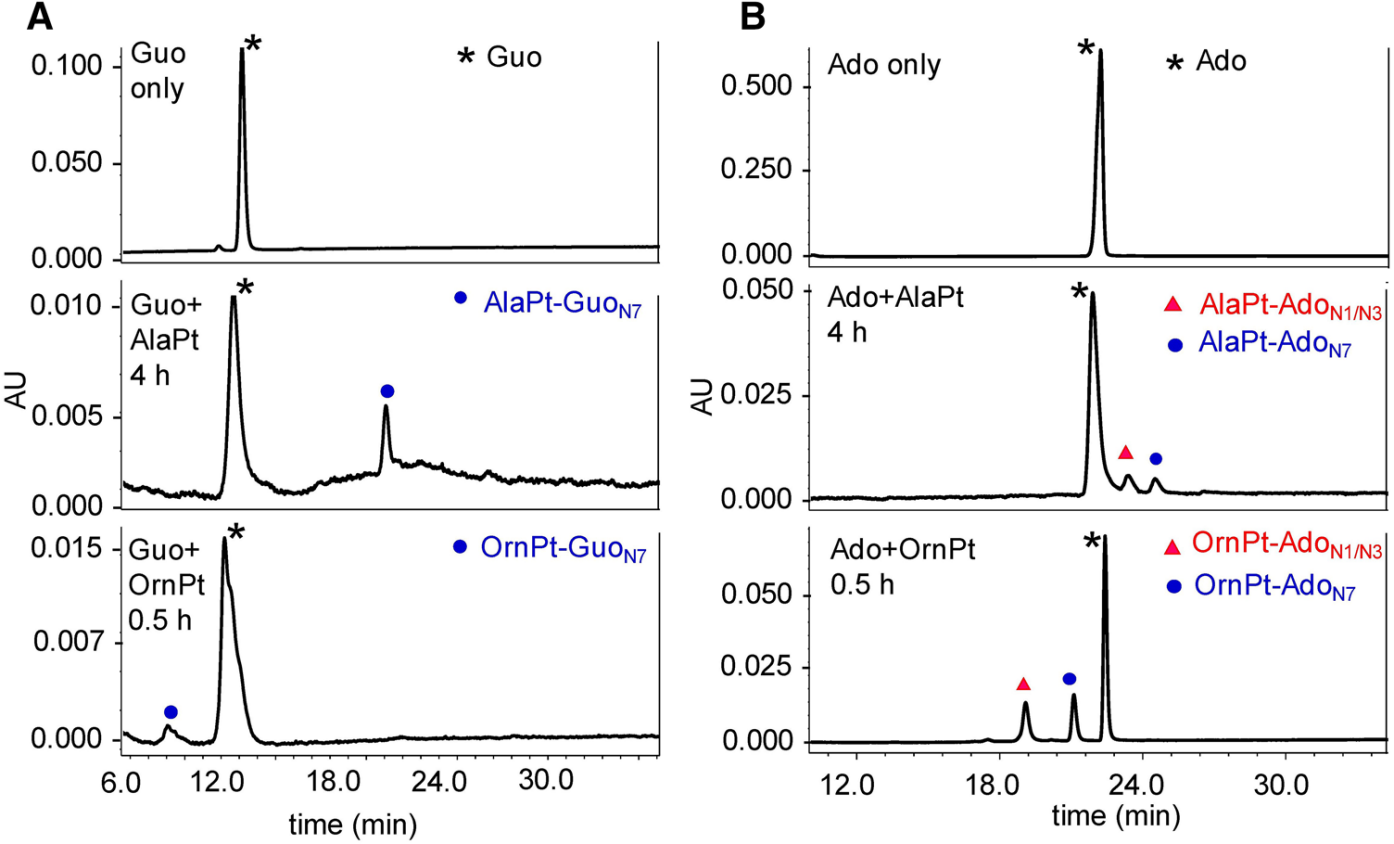
HPLC analysis (C18) of platination products. The AAPt-Guo (**a**) or AAPt-Ado (**b**) adduct profiles following reactions with monoaquated AlaPt or monoaquated OrnPt are shown. The peaks are labeled as follows; black asterisks for free nucleosides, blue circles for N7 platination adducts, and red triangles for N1/N3 platination adducts. The reaction times indicated are when visible amounts of products are formed. The controls (unreacted Guo or Ado) are shown as the top traces in **a** and **b**

The reactions of (deoxy)nucleosides with the monoaquated AAPt compounds result in the formation of one or more adducts with longer (AlaPt adducts) or shorter (OrnPt adducts) retention times on a C18 column relative to the unreacted (deoxy)nucleosides (Fig. 2). Only one AAPt-Guo adduct was expected based on the known preference for binding of the parent cisPt at the guanine N7 position [59, 60]. The monoaquated AlaPt reacts with Guo to form one major product, referred to as AlaPt-Guo_N7_ (in which the subscript is based on the expected coordination site), which has a longer retention time on C18 compared to Guo due to decreased polarity associated with the alanine ligand (Fig. 2a). Similarly, monoaquated OrnPt reacts with Guo to form one major product, OrnPt-Guo_N7_, which has a shorter retention time due to the polar nature of the ornithine side chain. In contrast, monoaquated AlaPt reacts with Ado to form two main products (distinct HPLC peaks), referred to as AlaPt-Ado_N1/N3_ and AlaPt-Ado_N7_ (Fig. 2b). The coordination sites were determined by NMR, which will be discussed below. These adducts have longer retention times compared to Ado. Monoaquated OrnPt also reacts with Ado to form two major products, OrnPt-Ado_N1/N3_ and OrnPt-Ado_N7_. These adducts have shorter retention times than Ado on C18.

**Fig. 3:**
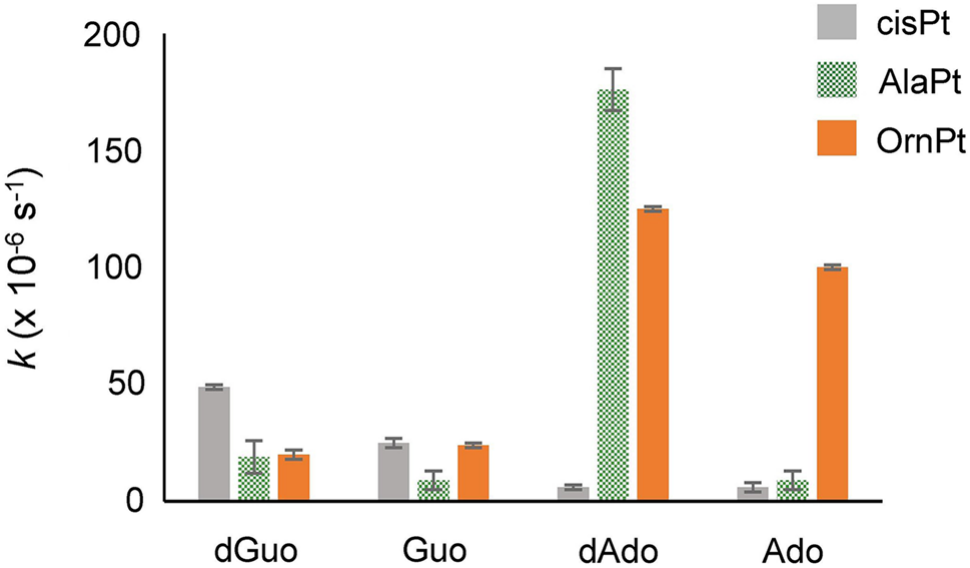
Comparisons of rate constants (*k*) for reaction of monoaquated cisPt (gray) and AAPt compounds (monoaquated AlaPt, green; monoaquated OrnPt, orange) with DNA/RNA purine nucleosides (dGuo, Guo, dAdo, and Ado) are shown

Kinetic rate constants (*k* values) were used to reveal the preferred target nucleoside of each AAPt compound relative to cisPt. Under pseudo-first-order reaction conditions for the monoaquated AAPt (or cisPt) and purine nucleosides, the peak areas of the unreacted nucleosides were monitored by HPLC and observed to decrease with time (Fig. S8). The rate of decrease of the peak area corresponding to the unreacted nucleoside (Ado, Guo, dAdo, or dGuo) varies, depending on the identity of the platinum compound. The HPLC calibration curves show a linear relationship between the nucleoside concentration and normalized peak area (Fig. S9). Therefore, the peak area of the nucleoside at each time point can be integrated quantitatively, plotted, and fitted to an exponential decay equation (Eq. S1) to extract the pseudo-first-order rate constant (*k*). The *k* values for each AAPt analog with DNA/RNA nucleosides were obtained (summarized in Table 1 and shown graphically in Fig. 3).

**Table 1:** Pseudo-first-order rate constants, *k* (μs^−1^)

	dGuo	Guo	dAdo	Ado
cisPt^a^	49 ± 1	25 ± 2	6 ± 1	6 ± 2
AlaPt^a^	19 ± 7	9 ± 4	176 ± 9	9 ± 4
OrnPt^a^	20 ± 1	24 ± 1	125 ± 1	100 ± 1

Triplicate runs in 25 mM Na_2_HPO_4_/NaH_2_PO_4_ buffer (pH 7) at 37 °C

^a^Monoaquated compounds

CisPt is known to preferentially target dGuo over other residues of DNA [4, 26, 60, 61]. This selectivity was confirmed here with the nucleoside level kinetic studies, in which the highest reaction rate constant (*k*) of aquated cisPt is with dGuo (49 × 10^−6^ s^−1^) (Table 1). The reactivity of monoaquated cisPt with Guo, dAdo, and Ado is lower than dGuo by approximately two-fold or more. The AAPt compounds display a wide range of reactivities as shown in Fig. 3. The highest reactivity of monoaquated AlaPt is with dAdo (176 × 10^−6^ s^−1^), which is nine- to 20-fold higher than that of reactions with Guo and dGuo, and demonstrates that monoaquated AlaPt prefers dAdo. The reactivity of monoaquated AlaPt is strongly influenced by both the identity of the purine base as well the functional group at the 2′ position (e.g., H or OH), with a preference for dAdo over Ado. The highest reaction rates for OrnPt are with dAdo and Ado (125 × 10^−6^ s^−1^ and 100 × 10^−6^ s^−1^, respectively). These rates of reactions for monoaquated OrnPt with Ado/dAdo are up to six-fold higher than reactions with Guo/dGuo (Fig. 3), demonstrating its preference for the adenine moiety. Monoaquated AlaPt and OrnPt both exhibit altered reactivity [25] and different nucleoside preferences compared to monoaquated cisPt. These differences suggest that the mode of amino acid coordination (e.g., N vs. O) plays a role in modulating the rate of reactivity. While cisPt only has only (N,N) type of binding, the amino acid complexes can have (N,O) or (N,N) coordination. Therefore, the next set of experiments addressed the adduct structures and how the amino acids influence the formation of different types of adducts.

### Characterization of AAPt-nucleoside adducts

When cisPt coordinates with DNA, it predominantly platinates the N7 position of dGuo [60, 62]. In this work, we sought to determine the binding site preferences and to characterize the types of adducts formed by AAPt compounds. The structures and corresponding chemical properties of various AAPt compounds may alter the preferred sites of reactivity versus those targeted by cisPt.

Platinum-based compounds may form intrastrand or interstrand adducts, depending on whether they coordinate to the same strand or two strands, respectively [1, 61]. Adducts can also be defined as monofunctional, bifunctional, or doubly platinated, depending on the platinum:nucleoside ratio (i.e., 1:1, 1:2, or 2:1, respectively). The adduct types also vary, depending on the site of platination on the nucleobase (e.g., N1, N3, or N7). In this work, mass spectrometry was used to determine the composition and type (e.g., mono- or bifunctional) of adducts formed by the platinum-based compounds, and ^1^H-NMR spectroscopy was used for characterization of the platination sites on the nucleoside.

**Fig. 4:**
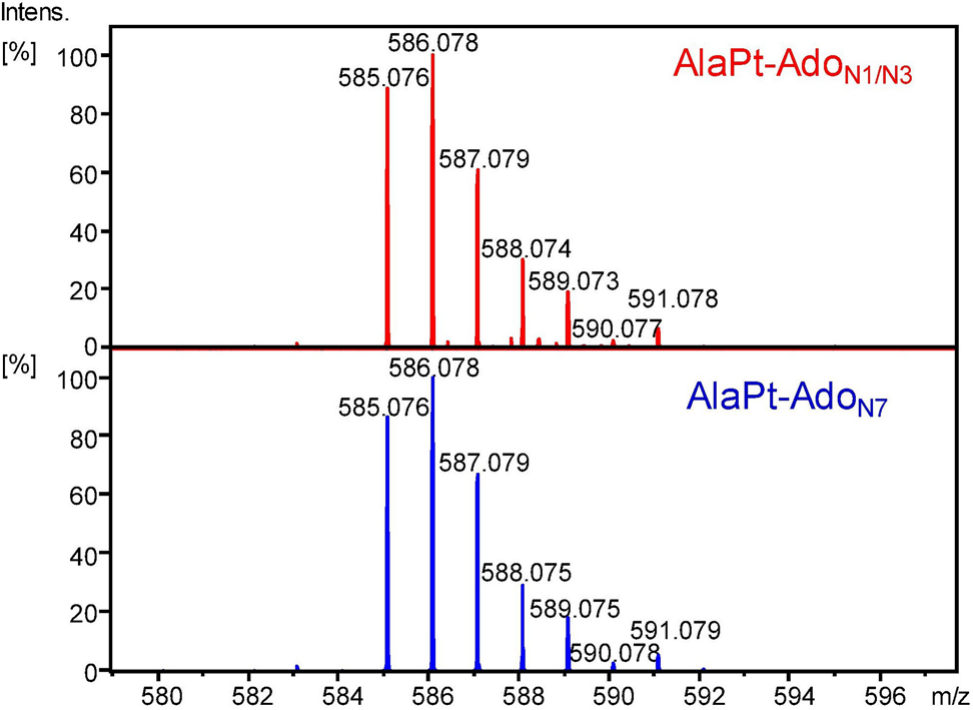
Mass spectra of the AlaPt-Ado_N1/N3_ and AlaPt-Ado_N7_ fractions. For both fractions, the monofunctional adduct species [Pt(Ado)(Ala)(Cl) + H]^+^ is observed with the predicted isotopic distribution

Mass analysis of the compounds in the two fractions obtained from HPLC C18 separation (Fig. 2), referred to as AlaPt-Ado_N1/N3_ and AlaPt-Ado_N7_, reveals that they have the same mass, charge, and isotopic distribution, corresponding to a monofunctional adduct, [Pt(Ado)(Ala)(Cl) + H]^+^ (Fig. 4). Because they have the same mass, the two adducts are constitutional isomers that may vary at the site of platination (N1, N3, or N7). For the geometric isomers, we expect that the structures shown in Scheme 1 are preferred because of the *trans* effect [63], but these structures still need to be confirmed experimentally. In addition, it would be of interest to understand how the binding types of platinum-based compounds (i.e., (N,N) vs. (N,O)) impact the structure of adducts and their formations. Other species formed by the loss and addition of neutral molecules are observed (Fig. S10). These species include [Pt(Ado)(Ala)(NH_3_)]^+^, [Pt(Ado)(Ala)(CH_3_CO_2_H)]^+^, and [Pt(Ado)(Ala)(CH_3_CN)]^+^, which form as a result of the monofunctional adduct losing HCl and generating a complex with NH_3_, CH_3_CO_2_H, or CH_3_CN from the HPLC buffer, respectively. Due to the soft ionization conditions offered by ESI, it is not surprising to observe adduction of NH_3_ [64].

**Fig. 5:**
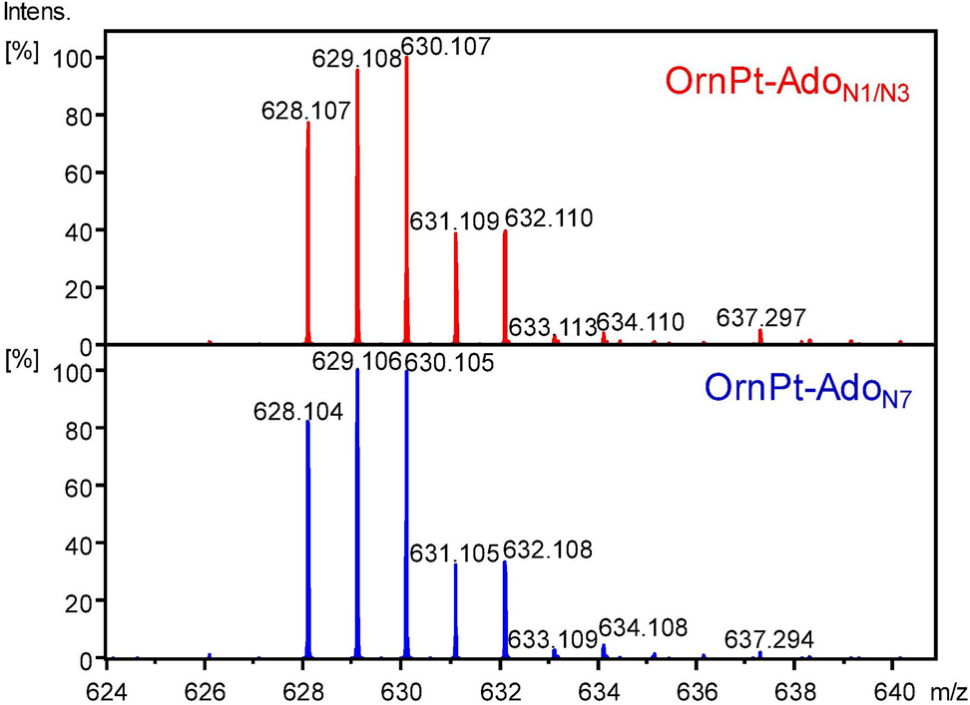
Mass spectra of the OrnPt-Ado_N1/N3_ and OrnPt-Ado_N7_ fractions. For both fractions, the monofunctional adduct species [Pt(Ado)(Orn)(Cl)]^+^ is observed with the predicted isotopic distribution

Similar to that found for the reactions of AlaPt with Ado, compounds in the HPLC fractions referred to as OrnPt-Ado_N1/N3_ and OrnPt-Ado_N7_ have the same mass, charge, and isotopic distribution corresponding to a monofunctional adduct [Pt(Ado)(Orn)(Cl)]^+^ (Fig. 5), i.e., the two adducts are constitutional isomers with variation at the site of coordination (e.g., N1, N3, or N7) or varying geometries as discussed for AlaPt. Species assigned as [Pt(Ado)(Orn)(NH_3_)–H]^+^ and [Pt(Ado)(Orn)(CH_3_COOH)–H]^+^ are also observed, suggesting that ligand substitution of one of the chlorido ligands is facile in solution (Fig. S10).

NMR analysis was used to assign the platination sites of the adducts [55]. First, HSQC spectroscopy was used to assign the H2 and H8 protons of Ado, OrnPt-Ado_N7_, and OrnPt-Ado_N1/N3_ (Fig. S11). Subsequently, ^1^H-NMR spectroscopy was used to assign the specific platination sites in the AlaPt-Ado and OrnPt-Ado adducts (Figs. S12 and S13). Compared to the chemical shifts of the H2 and H8 protons of Ado, the corresponding peaks for the species assigned as AlaPt-Ado_N1/N3_ are shifted downfield by 0.4 and 0.1 ppm, respectively (Table 2). The greater change in chemical shift of H2 compared to H8 suggests platination at the N1 position in AlaPt-Ado_N1/N3_ because of its proximity to H2 [59, 65]. However, we cannot rule out platination at N3 of Ado, because that adduct would also cause a downfield shift of H2. ^1^H-NMR analysis of AlaPt-Ado_N7_ shows the peaks assigned as H2 and H8 shifting downfield by 0.1 and 0.5 ppm, respectively, suggesting that platination occurs at the N7 position for this adduct. The analysis of platination sites on the monoaquated OrnPt adducts is similar to that of the AlaPt adducts (Fig. S13). The larger chemical shift change of the H2 peak is consistent with N1/N3 platination of the HPLC fraction assigned as OrnPt-Ado_N1/N3_, whereas OrnPt-Ado_N7_ is assigned as having N7 platination (Table 3).

**Table 2:** Downfield shifts of protons (in ppm) of AlaPt-Ado adducts relative to Ado protons

	H2	H8
AlaPt-Ado_N1/N3_^a^	0.4	0.1
AlaPt-Ado_N7_^b^	0.1	0.5

^a^Monofunctional [Pt(Ado)(Ala)(Cl)] with N1/N3 platination

^b^Monofunctional [Pt(Ado)(Ala)(Cl)] with N7 platination

**Table 3:** Downfield shifts of protons (in ppm) of OrnPt-Ado adducts relative to Ado protons

	H2	H8
OrnPt-Ado_N1/N3_^a^	0.5	0.1
OrnPt-Ado_N7_^b^	0.1	0.5

^a^Monofunctional [Pt(Ado)(Orn)(Cl)] with N1/N3 platination

^b^Monofunctional [Pt(Ado)(Orn)(Cl)] with N7 platination

**Fig. 6:**
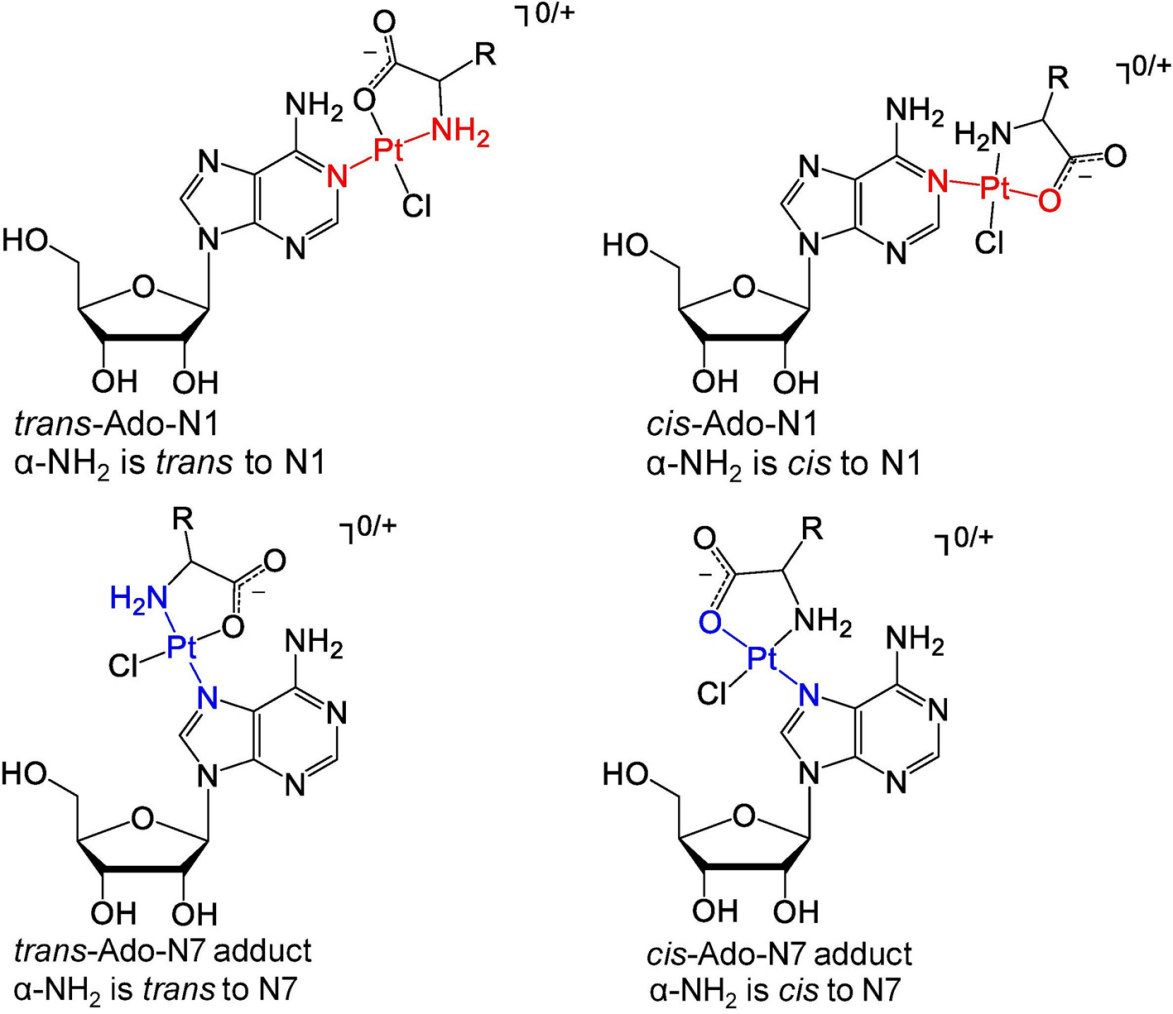
Representative geometric and constitutional isomers of AAPt-Ado adducts with platination at the N1 or N7 positions (N3 platination is also possible). The adducts may have different orientations to position functional groups for favorable interactions such as hydrogen bonding or to avoid steric clash

In addition to the NMR data, the kinetic *trans* effect and previously reported theoretical calculations were used to propose the structures of the adducts [63, 66]. The adducts likely have several structural orientations that allow maximum stability. We propose that AlaPt-Ado and OrnPt-Ado adducts assume the *trans* orientations in which the α-NH_2_ is *trans* to the Ado platination site (N1, N3, or N7) (Fig. 6). These orientations allow the AAPt compounds to coordinate to the nucleoside such that the α-amino group and the platinated nitrogen on the nucleoside are *trans* to each other. These structures can be used to explain the nucleoside preferences of the platinum-based compounds. The selectivity of cisPt for dGuo has been attributed predominantly to a strong hydrogen bond between the cisPt ammine ligand and the 6-oxo group of Guo [36]. In contrast, the selectivity of AlaPt and OrnPt for Ado/dAdo can be explained by a hydrogen-bonding interaction unique to the AAPt compounds. The *trans* adduct structures (e.g., *trans*-Ado-N1 or *trans*-Ado-N7 in Fig. 6) would position the 6-amine of Ado to form a hydrogen bond with the carboxylate ligand of the AAPt compound. Such interactions would stabilize the Ado adduct, but are not available for Guo, therefore, leading to lower reactivities of Guo/dGuo with the two AAPt compounds studied here. The formation of N3 platination products is less likely due to lack of a stabilizing hydrogen bond with the position 6 functional group of the purine (Fig. S14). OrnPt could potentially form a more stabilized *cis* adduct structure with Guo at the N7 position (*cis*-Guo-N7) through hydrogen bonding of the 6-oxo group and the amine side chain (Fig. S15).

### Differential activation of AlaPt-Ado and OrnPt-Ado isomers toward glycosidic bond cleavage

Stability of the glycosidic bond is important in maintaining the integrity of nucleic acids. Unstable glycosidic bonds accelerate depurination and formation of abasic sites on nucleic acids, which may ultimately contribute to cell cytotoxicity [67]. Destabilization of the glycosidic bond can be caused by a change in chemical composition of the nucleoside due to certain modifications such as methylation [45, 47, 68]. Previous evaluation of the effects of cisPt modification on guanosine residues indicated that platination has no significant effect on glycosidic bond stability [52]. Because AAPt compounds have altered reactivity and platinate Ado residues at more than one site, we investigated the relative glycosidic bond stabilities of Ado residues after modifications with these AAPt compounds. Glycosidic bond survival yield analysis employing ER-CID tandem mass spectrometry has been used previously to study relative stabilities of the glycosidic bonds in the context of cationization and protonation of purine (deoxy)nucleosides [46, 50, 69, 70]. Glycosidic bond survival yield of the two AlaPt-Ado adducts (referred to as N1/N3 and N7, depending on the platination site) was compared to determine their relative glycosidic bond stabilities. Because the adducts are structural isomers, they have an equal number of atoms and degrees of freedom, and thus internal energy and dissociation lifetime effects on the fragmentation should be very similar such that differences in the survival yield can be directly correlated to glycosidic bond stability. The two OrnPt-Ado adducts were compared in a similar fashion.

**Fig. 7:**
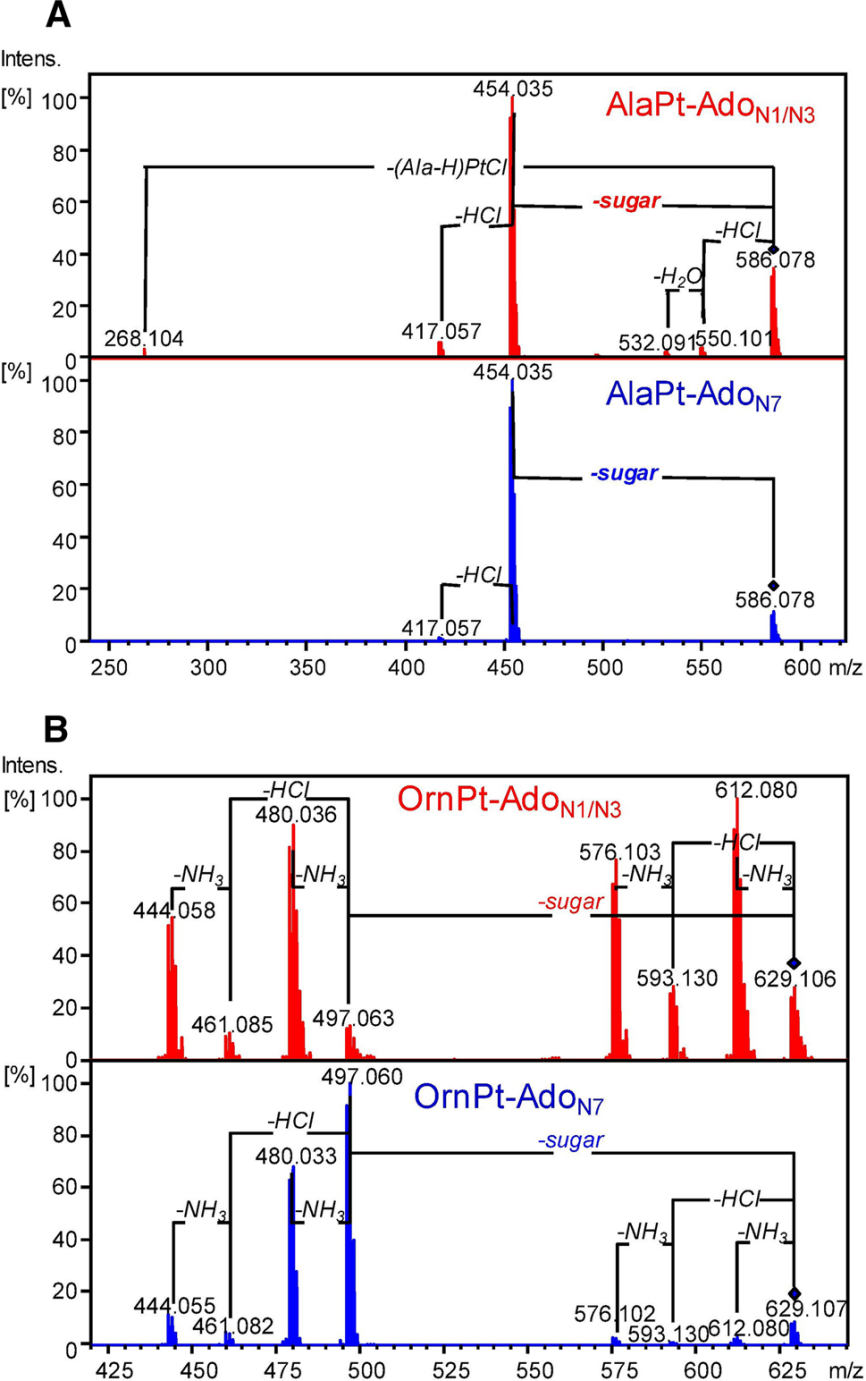
CID fragmentation patterns of AAPt-Ado adducts. **a** AlaPt-Ado_N1_ (or AlaPt-Ado_N3_) and AlaPt-Ado_N7_ mass spectra are shown. **b** OrnPt-Ado_N1_ (or OrnPt-Ado_N3_) and OrnPt-Ado_N7_ fragmentation patterns are given

The CID fragmentation patterns of AlaPt-Ado_N1/N3_ and AlaPt-Ado_N7_ were determined first. Despite having different platination sites, the N1/N3 and N7 adducts of AlaPt-Ado produce comparable fragmentation patterns with a major fragment (*m*/*z*: 454.036 Da) formed by loss of the ribose sugar (132.042 Da) via glycosidic bond cleavage following proton transfer of H2′ to the nucleobase (Fig. 7a) [46]. Minor neutral losses of HCl, H_2_O, and [Pt(Ala)(Cl)–H] from AlaPt-Ado_N1/N3_ (*m*/*z*: 550.101, 532.091 and 268.104 Da, respectively) are also observed.

In contrast to the AlaPt-Ado adducts, fragmentation patterns of the OrnPt-Ado adducts vary between the isomers, despite being subjected to similar dissociation energies (Fig. 7b). Two groups of fragments are observed in each CID mass spectrum. The first group contains fragments formed by loss of neutral molecules of NH_3_, HCl, and (NH_3_ + HCl) from OrnPt-Ado (*m*/*z*: 612.080, 593.130, and 576.103 Da, respectively); the second group corresponds to fragments involving both glycosidic bond cleavage ions resulting in loss of the ribose sugar and the same neutral losses (*m*/*z*: 497.063, 480.036, 461.085, and 444.058 Da, respectively). For OrnPt-Ado_N1/N3_, both groups have approximately the same intensity, whereas for OrnPt-Ado_N7_, the second group of fragments dominates. This result indicates a difference in the overall stability of the glycosidic bonds of these two OrnPt-Ado isomers.

**Fig. 8:**
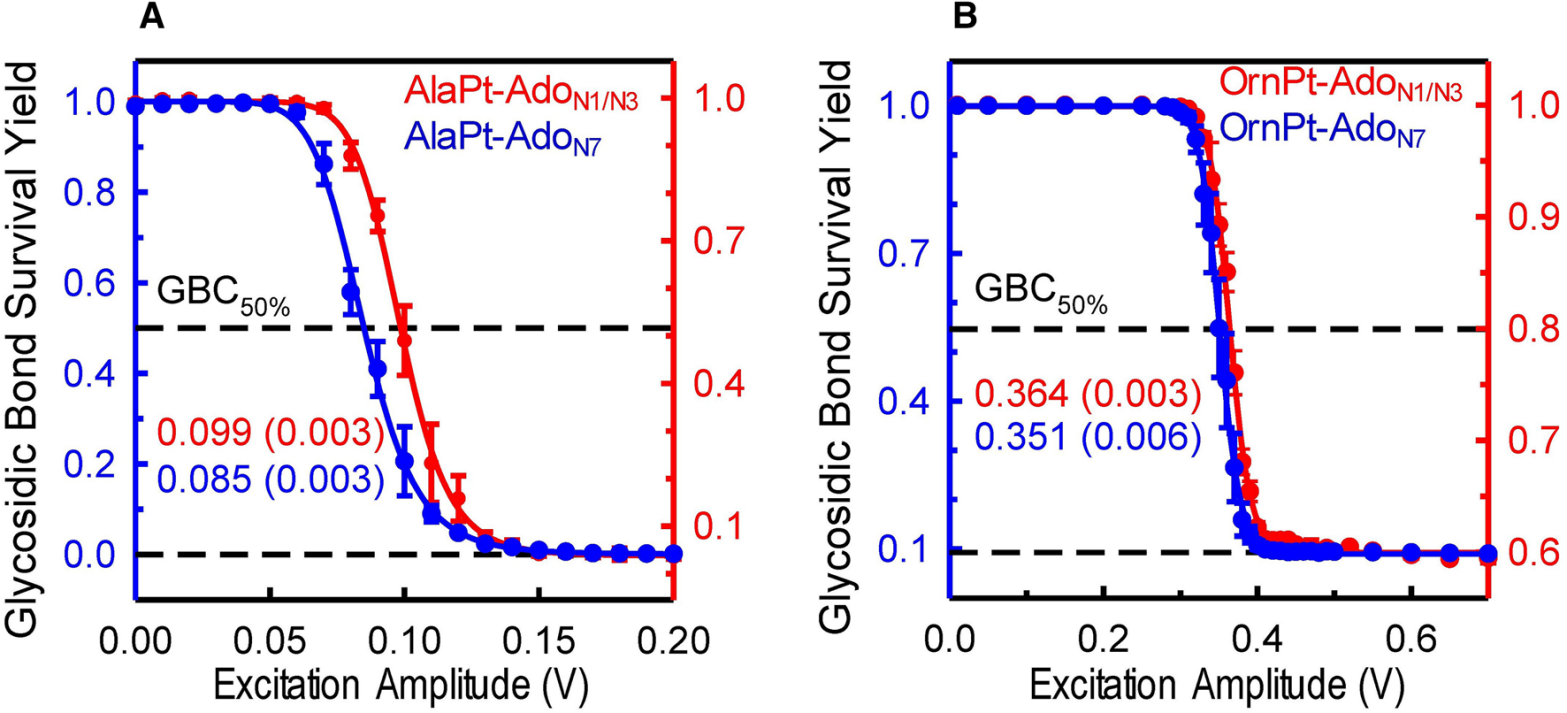
Glycosidic bond survival yield curves of platinum adducts. Glycosidic bond survival yield curves of **a** AlaPt-Ado isomers (AlaPt-Ado_N1/N3_ in red and AlaPt-Ado_N7_ in blue) and **b** OrnPt-Ado isomers (OrnPt-Ado_N1/N3_ in red and OrnPt-Ado_N7_ in blue) are shown

Survival yield analysis is a method that monitors fragmentation patterns of ions as a function of excitation voltage [57]. In the next set of experiments, survival yield analysis was used to investigate the stability of the glycosidic bonds in AAPt-nucleoside adducts isomers. Fits to the survival yield curves of the AlaPt-Ado isomers produce GBC_50%_ values for AlaPt-Ado_N1/N3_ and AlaPt-Ado_N7_ of 0.099 V and 0.085 V, respectively (Fig. 8a). Platination in both isomers activates dissociation primarily through cleavage of the glycosidic bond with 95% and 100% of the fragmentation of AlaPt-Ado_N1/N3_ and AlaPt-Ado_N7_ occurring via glycosidic bond cleavage, respectively, i.e., neutral losses account for 5% or less of the dissociation behavior for both AlaPt-Ado isomers. Trends in the GBC_50%_ values indicate that N7 platination is more activating than N1/N3 platination. The GBC_50%_ values determined for OrnPt-Ado_N1/N3_ and OrnPt-Ado_N7_ are much larger, 0.364 V and 0.351 V, respectively. The trend in these values again indicates that N7 platination activates the glycosidic bond more effectively than N1/N3 binding of platinum. Furthermore, both OrnPt-Ado adducts exhibit significant competition between glycosidic bond cleavage and neutral loss pathways. Indeed, neutral loss pathways account for 60% and 10% of dissociation for OrnPt-Ado_N1/N3_ and OrnPt-Ado_N7_ adducts, respectively. Because AlaPt and OrnPt-Ado differ in size, direct comparison of GBC_50%_ values may be problematic, but the large shifts in these values determined for the AlaPt-Ado vs. OrnPt-Ado adducts strongly suggest that binding of AlaPt activates the glycosidic bond more effectively than OrnPt binding. The increased competition with the neutral loss pathways in OrnPt-Ado compared to AlaPt-Ado adducts also supports this conclusion.

The difference in stability between the adduct isomers suggests that two factors can influence the glycosidic bond stability, the type of amino acid ligand linked to the platinum center, and the site at which the AAPt coordinates with the nucleoside. Platination by monoaquated AlaPt results in adduct isomers that have relatively lower glycosidic bond stabilities than adduct isomers formed by monoaquated OrnPt. The fact that N7 platination by AlaPt and OrnPt results in lower adduct glycosidic bond stability compared to N1 (or N3) platination suggests that the closer the proximity of the platination site to the ribose sugar, the greater the destabilization of the glycosidic bond. Protonation or platination modifications on the nucleoside can accelerate the depurination reaction by reducing the transition state energy [52]. The ligands of AlaPt or OrnPt likely cause electron-density withdrawal at the N9 position in a manner that is dependent on proximity of the platinum atom. Further tandem mass spectrometry and spectroscopic studies, including computational analysis, are ongoing in an effort to elucidate detailed mechanisms for glycosidic bond cleavage of the N7 and N1/N3 AlaPt and OrnPt adduct species, as done previously with both standard and modified nucleosides [42, 46, 68].

## Conclusions

CisPt preferentially binds with dGuo residues, which plays a role in its anticancer activity. Nonetheless, cisPt is not effective against all forms of cancer and there are drawbacks such as DNA repair of the resulting cisPt adducts that limit its effectiveness. Formation of adducts at sites not targeted by cisPt could be important for alternative cytotoxicity pathways in tumor cells. Finding platinum-based compounds that preferentially platinate alternative non-cisPt targets has been an attractive goal, but has yielded little success to date. In this report, platinum-based compounds OrnPt and AlaPt exhibit template-independent kinetic preferences for Ado/dAdo residues. OrnPt exhibits a greater than five-fold increase in reactivity with Ado/dAdo residues over Guo/dGuo, and AlaPt has over nine-fold higher selectivity for dAdo over all other purines. The AAPt compounds show significant differences in their reactivity with nucleosides compared to cisPt. Thus, the two AAPt compounds studied here have altered selectivity compared to cisPt and are, therefore, candidates for an alternative pathway of cytotoxicity that could involve targeting dAdo residues of DNA as well as Ado residues of RNA. The preference of AlaPt for dAdo could be potentially advantageous for the development of compounds that can target DNA over RNA. Since platination of biomolecules such as proteins are often overlooked [19, 22, 23], AAPt compounds may also have reactivity that extends beyond the current scope of targets. Mass spectrometry-based proteomics have been used to study cross-links of DNA and proteins caused by cisPt [71], and these methods could also be applied to characterize possible RNA-protein cross-links induced by AAPt.

In contrast to cisPt, AlaPt and OrnPt were observed to coordinate to multiple sites on Ado/dAdo residues, specifically the N1/N3 and N7 positions of the nucleobases. The cisPt adducts occur predominantly at the N7 position of Guo residues. Because the AAPt compounds may coordinate to varying positions of the nucleoside, they could potentially be used to identify solvent accessible sites, especially in secondary structures of RNA compared to DNA. The AAPt compounds can complement existing chemical probes, including cisplatin, that have been used to identify accessible sites in nucleic acids within complex biological environments [14, 25]. While the current experiments are at the nucleoside level, the ability of AAPt compounds to identify and form adducts at sites in folded RNA has been observed previously [25]. These adducts may also disrupt Watson–Crick base pairing involving N1 of (d)Ado in both nucleic acids or other interactions involving N3 or N7 in RNA. Such altered reactivity and expansion of platination target sites beyond dGuo may be potentially important in causing inhibition of cellular pathways that is not observed with cisPt.

Enzymatic cleavage of Ado glycosidic bonds in RNA and subsequent depurination are lethal to cells [53, 72]. Alternatively, cleavage though activation of glycosidic bonds could lead to fragmentation of nucleic acids [41–44]. In this work, glycosidic bond survival yield analysis was applied to determine the impact of AAPt coordination on the relative glycosidic bond strength of an RNA-based nucleoside, Ado. When either AlaPt or OrnPt binds at the N7 position of Ado, they destabilize the glycosidic bond to a greater extent than when coordination occurs at the N1/N3 position. Overall, AlaPt almost exclusively activates the resulting AlaPt-Ado adduct via cleavage of the glycosidic bond. In contrast, OrnPt is less activating of the glycosidic bonds such that fragmentation of OrnPt-Ado also involves other neutral loss pathways. Such activation of AAPt-Ado adducts is particularly important considering that cisPt is reported to have no destabilizing effect on the glycosidic bonds of target dGuo residues [52]. Within a biological context, AlaPt and OrnPt could potentially exert destabilization of glycosidic bonds in nucleic acid residues and lead to abasic sites. Formation of abasic sites, especially in RNA, could possibly trigger apoptosis, as well as provide an alternative pathway of cytotoxicity that circumvents cisPt resistance. The variation in the influence of AlaPt and OrnPt on Ado also highlights the importance of structure for other classes of platinum-based drugs in determining their preferred target residues and impact on adduct stability. In future studies, we will employ other techniques such as X-ray crystallography to differentiate between N1 and N3 adducts of amino acid-linked platinum compounds and possibly (N,O) vs. (N,N) type coordination. Ongoing studies are also being carried out to determine how the local environment and sequence contexts impact kinetics of AAPt reactions with oligonucleotides, along with cellular studies with various cell lines. By combining cell studies with tandem mass spectrometry studies, we can further explore the importance of platination sites on adduct stability, biomolecular interactions, and downstream biological functions. The method of survival yield analysis is applicable to other fields such as proteomics in which the effects of modifications (e.g., platination, alkylation) on the stability of peptides could be determined.

## Electronic supplementary material

Below is the link to the electronic supplementary material.

Supplementary material 1 (PDF 5675 kb)

## Abbreviations

AAPt: Amino acid-linked platinum(II) compounds
cisPt: *cis*-diamminedichloridoplatinum(II)
AlaPt: Alanine-linked platinum(II) compound
OrnPt: Ornithine-linked platinum(II) compound
Guo: Guanosine
dGuo: 2′-Deoxyguanosine
Ado: Adenosine
dAdo: 2′-Deoxyadenosine
