# Supplementary material for: Amino acid-linked platinum(II) compounds: non-canonical nucleoside preferences and influence on glycosidic bond stabilities
Source: J Biol Inorg Chem. 2019 Jul 29;24(7):985–97. doi: 10.1007/s00775-019-01693-y (PMC6806012; doi:10.1007/s00775-019-01693-y)
Supplement: Supplementary file 1 — Supplementary material 1 (PDF 5675 kb) [file 775_2019_1693_MOESM1_ESM.pdf]

Supporting information

Amino acid-linked platinum(II) compounds: Non-canonical nucleoside preferences and influence on glycosidic bond stabilities

Bett Kimutai, C. C. He, Andrew Roberts, Marcel L. Jones, Xun Bao, Jun Jiang, Zhihua Yang, M.T. Rodgers, and Christine S. Chow*

Department of Chemistry, Wayne State University, Detroit, Michigan 48202, United States.

Table of contents

Figure S1	^{1}H-NMR profile of L-alanine and AlaPt	p. S3
Figure S2	^{13}C-NMR profile of L-alanine and AlaPt	p. S3
Figure S3	^{195}Pt-NMR profile of potassium tetrachloroplatinate(II) and AlaPt	p. S4
Figure S4	Mass analysis of AlaPt	p. S4
Figure S5	^{1}H-NMR spectra of L-ornithine and OrnPt	p. S5
Figure S6	Mass analysis of OrnPt	p. S5
Figure S7	Crystal structure of OrnPt	p. S6
Figure S8	Reaction rates of AlaPt and OrnPt with purine nucleosides	p. S8
Figure S9	HPLC calibration curves of adenosine and guanosine	p. S9
Figure S10	Mass analysis of the AAPt-Nuo fractions	p. S10
Figure S11	2D heteronuclear single quantum correlation (HSQC) spectroscopy of OrnPt-Ado$_{N7}$ and OrnPt-Ado$_{N1/N3}$	p. S11
Figure S12	Aromatic region of 1D ^{1}H-NMR spectra of AlaPt-Ado adducts	p. S12
Figure S13	Aromatic region of ^{1}H-NMR spectra of OrnPt-Ado adducts	p. S12
Figure S14	Possible isomers and orientations of AAPt-purine adducts	p. S13
Figure S15	OrnPt-Guo$_{N7}$ with two stabilizing hydrogen bonds	p. S14

Characterization of AAPt compounds. AlaPt and OrnPt were characterized by using ^{1}H-NMR spectroscopy (**Figs. S1** and **S3**) and mass spectrometry analysis (**Figs. S2** and **S4**). The ^{1}H-NMR profile of AlaPt was analyzed in comparison to the ^{1}H-NMR profile of L-alanine (**Fig. S1**). The H_{α} proton peak of alanine has a chemical shift 3.61 ppm; whereas, the corresponding H_{α} peak for AlaPt is shifted upfield by 0.11 ppm (δ 3.50 ppm). The H_{β} proton peak of L-alanine has a chemical shift of 1.31 ppm; whereas, the corresponding H_{β} peak for AlaPt is shifted slightly upfield by 0.03 ppm (δ 1.28 ppm). In the ^{13}C-NMR spectrum, the carboxylate carbon peak for alanine has a chemical shift of 176 ppm; whereas, the corresponding peak for AlaPt is at 193 ppm (**Fig. S2**). The C_{α} and C_{β} are shifted less (from 50 to 57 ppm and 16 to 20 ppm, respectively) because they are further away from the platinum center. In the ^{195}Pt-NMR spectrum, the ^{195}Pt chemical shift of potassium tetrachloroplatinate(II) is compared to that of AlaPt (**Fig. S3**). Potassium tetrachloroplatinate(II) and AlaPt have ^{195}Pt chemical shifts of -1607 and -1639 ppm, respectively.

The ^{1}H-NMR spectrum of OrnPt was also analyzed in comparison to that of L-ornithine (**Fig. S5**). The chemical shift of the L-ornithine H_{α} proton peak is 3.60 ppm. The close proximity of the metal center shifts the corresponding H_{α} proton for OrnPt upfield to 3.50 ppm. The platinum-induced chemical shift is subtle for the H_{β} and H_{γ} protons, likely because they are further away from the metal center.

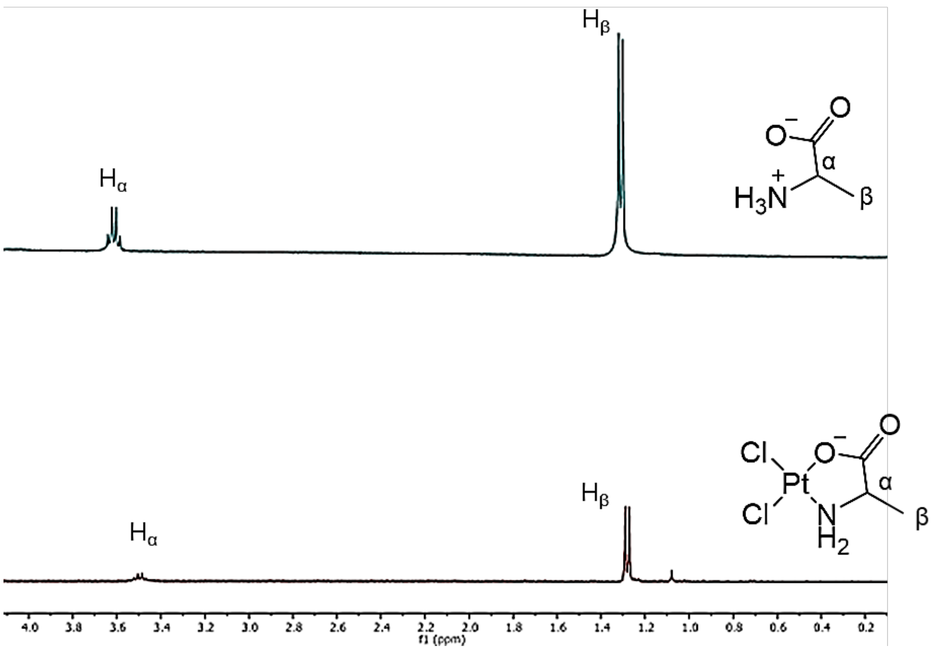

Figure S1. The ^{1}H-NMR profile of L-alanine (top) and AlaPt (bottom). For L-alanine; δ/ppm (^{1}H-NMR, D_2O, 400 MHz), 3.61 (1 H, q, J=7.7 Hz, =C$\underline{H}$$CH_3$), 1.31 (3 H, d, J=7.6 Hz, -C$\underline{H}_3$). For AlaPt; δ/ppm (^{1}H-NMR, D_2O, 400 MHz), 3.50 (1 H, q, J=7.1 Hz, =C$\underline{H}$$CH_3$), 1.28 (3 H, d, J=7.0 Hz, -C$\underline{H}_3$).

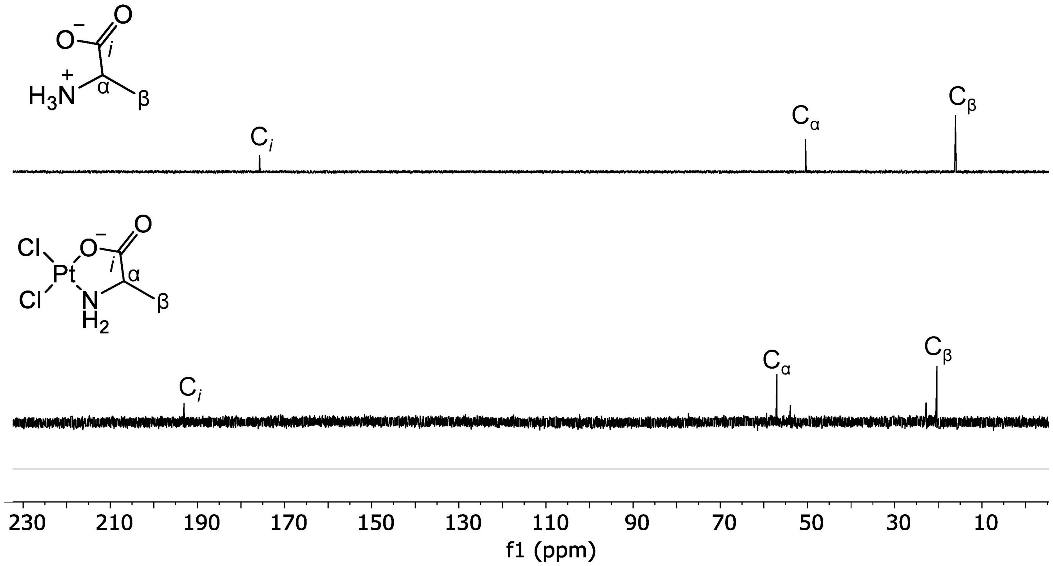

Figure S2. The ^{13}C-NMR profile of L-alanine (top) and AlaPt (bottom). For L-alanine; δ/ppm (^{13}C-NMR, D_2O, 400 MHz) 176 ((-$\underline{C}$OO)CH=), 50 (=$\underline{C}$$HCH_3$), 16 (=CH$\underline{C}H_3$); For AlaPt; δ/ppm (^{13}C-NMR, D_2O, 400 MHz) 193 ((-$\underline{C}$OO)CH=), 57 (=$\underline{C}$$HCH_3$), 20 (=CH$\underline{C}H_3$). The minor peaks are due to aquation of AlaPt which occurs in an aqueous environment.

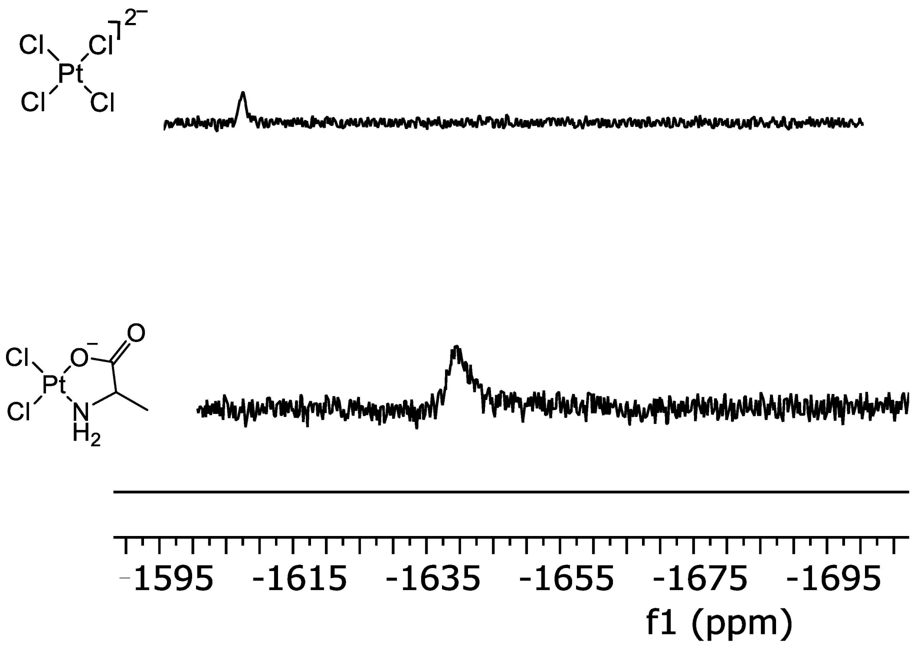

Figure S3. The ^{195}Pt-NMR profile of potassium tetrachloroplatinate(II) (top) and AlaPt (bottom). For potassium tetrachloroplatinate(II); δ/ppm (^{195}Pt-NMR, D_2O, 400 MHz) -1607 (<u>Pt</u>Cl_4); For AlaPt; δ/ppm (^{195}Pt-NMR, D_2O, 400 MHz) -1639 (<u>Pt</u>$Cl_2(NH_2)O$). The ^{195}Pt chemical shift for AlaPt is in the same region where peaks for other comparable platinum-based compounds are found [1-2].

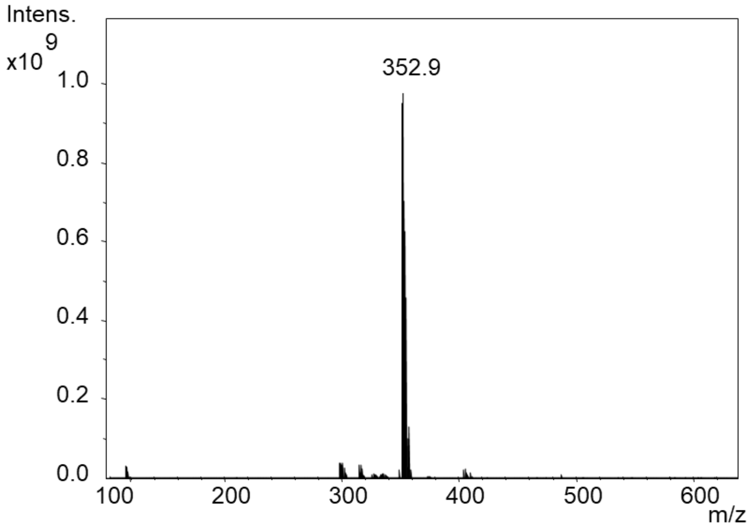

Figure S4. Mass analysis of AlaPt. *m/z* (electrospray ionization (ESI) mass spectrometry) 352.943 Da/e $[AlaPt]^-$; calculated exact mass 352.942 Da $[AlaPt]^-$; chemical formula $PtC_3H_6Cl_2NO_2$ ($[AlaPt]^-$, at neutral pH).

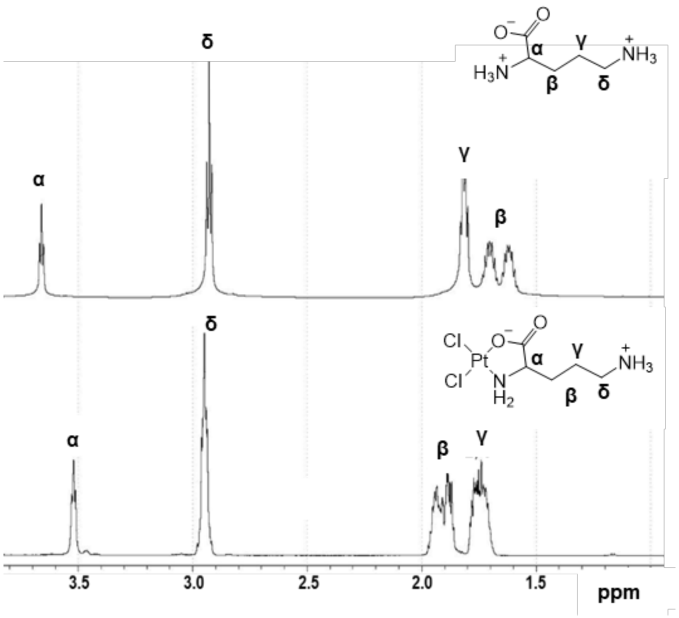

Figure S5. The ^{1}H-NMR spectra of L-ornithine (upper) and OrnPt (lower) as previously described [3]. For L-ornithine; δ/ppm (^{1}H-NMR, D_2O, 400 MHz), 3.63 (1 H, t, J=6.3 Hz, -C<u>H</u>(NH_3)CH_2-), 2.90 (2 H, t, J=8.0 Hz, -C<u>H</u>$_2$$NH_3$), 1.82 – 1.52 (4H, m, -CH(NH_3)C<u>H</u>$_2$C<u>H</u>$_2$-). For OrnPt; δ/ppm (^{1}H-NMR, D_2O, 400 MHz), 3.54 (1 H, t, J=6.3 Hz, -C<u>H</u>(NH_2)CH_2-), 2.89 (2 H, t, J=7.9 Hz, -C<u>H</u>$_2$$NH_3$), 1.84 – 1.69 (4H, m, -CH(NH_2)C<u>H</u>$_2$C<u>H</u>$_2$-).

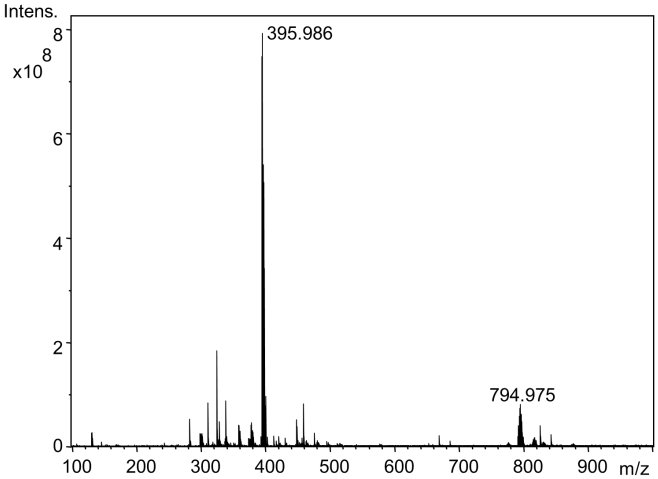

Figure S6. Mass analysis of OrnPt using FT-ICR MS: m/z (ESI-MS) 395.986 Da/e $[M\text{-}H]^{-}$; calculated exact mass 395.984 Da $[M\text{-}H]^{-}$; chemical formula $PtC_5H_{11}Cl_2N_2O_2$ $[OrnPt\text{-}H]^{-}$; at neutral pH); a dimer $[2(OrnPt)\text{-}H]^{-}$ is observed at 794.975 Da/e.

OrnPt was also characterized by solving its crystal structure through X-ray crystallography. The crystals were first grown through the slow evaporation method. A 10 mM concentration of OrnPt dissolved in double-distilled water (ddH_2O) was placed in a capped glass tube fitted with a needle-sized hole at the top. The tube was kept in the dark for 3 weeks at room temperature with minimal disturbance. Shiny yellow rod-like crystals were obtained. The biggest crystal was selected and the structure was solved using a Bruker D8 diffractometer (**Fig. S7**). The structure shows that the Pt center is coordinated with nitrogen and oxygen of the amino acid (N,O) binding mode.

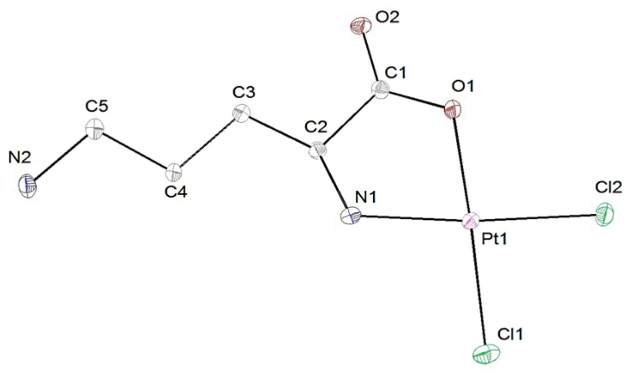

Figure S7. Crystal structure of OrnPt confirming a previously described (N_b,O_b) binding structure [3], in which the platinum maintains its square planar geometry in a five-membered chelation ring.

Fitting data into a kinetic equation. The pseudo-first order kinetics equation is derived as follows, in which $AAPt_{aq}$ is the monoaquated compound and Nuo is the nucleoside [4]:

$$\text{Nuo} + \text{AAPt}_{aq} \xrightarrow{k_{app}} \text{AAPt-Nuo adduct} + H_2O$$

Depending on the platinum-based compound, various charged species such as monoaquated or bisaquated species can be present in the solution and take part in the reaction [5]. The rate of reaction (V) is given by,

$$V = k_{app} [AAPt_{aq}] [Nuo]$$

in which, k_{app} is the apparent bimolecular rate constant. Under pseudo-first-order conditions, $[AAPt_{aq}] >> [Nuo]$ (50-fold excess of $AAPt_{aq}$ over Nuo) and remains approximately constant. Under these conditions, the rate constant, k, equals $k_{app}[AAPt_{aq}]$ and the rate (V) is given by,

$$-d[Nuo]/dt = k [Nuo]$$

By integration and rearrangement,

$$\ln[Nuo]_t = -kt + \ln[Nuo]_0$$

By rewriting the above equation in exponential form,

$$[Nuo]_t = e^{-kt} + [Nuo]_0 \qquad \textbf{(S1)}$$

in which $[Nuo]_t$ is the concentration of unreacted nucleoside at time t, and $[Nuo]_0$ is the initial concentration of unreacted nucleoside. When $[Nuo]_t$ is plotted against t, the decay factor is k. Experiments were performed in triplicate. The standard error was calculated by dividing the standard of the mean deviation by the number of replicates.

HPLC analysis of platination reaction of nucleosides. HPLC profiles were used to monitor the platination reactions over time (**Fig. S8**). The peak areas of the unreacted nucleosides are observed to decrease with time as expected, but with varying rates depending on the reactant nucleoside and the platinum-based compound used (monoaquated AlaPt or OrnPt)

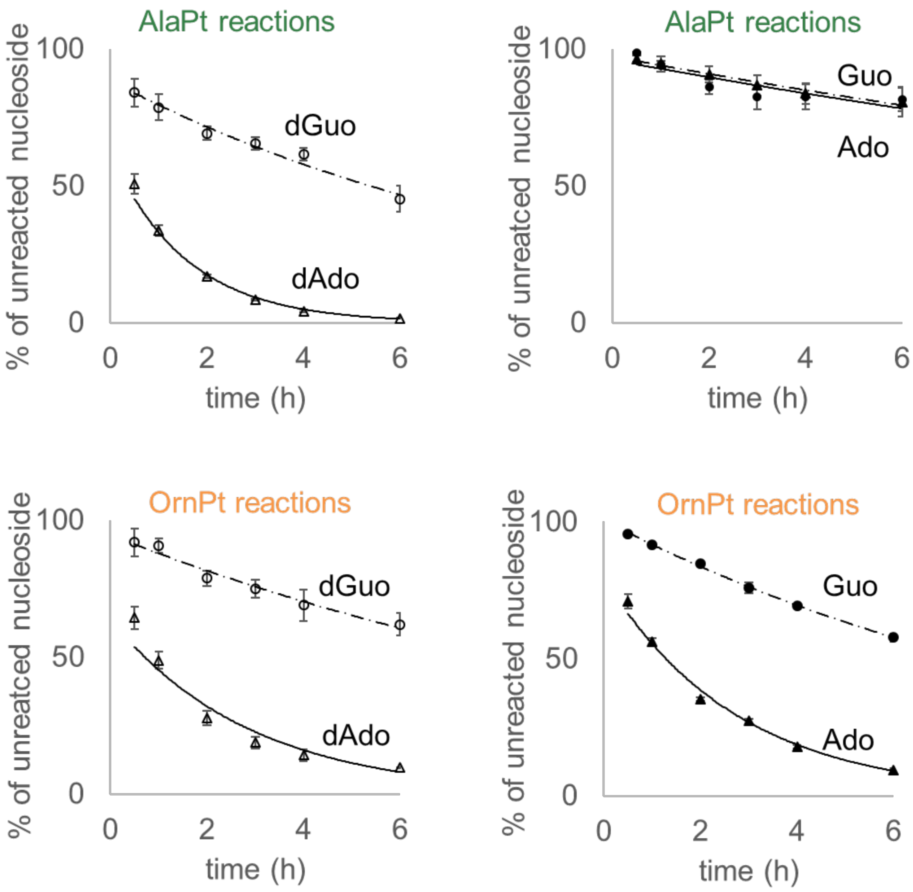

Figure S8. Reaction rates of AlaPt and OrnPt with the purine nucleosides. Representative kinetic traces of reactions of monoaquated AlaPt and monoaquated OrnPt with the purine nucleosides showing the fraction of the unreacted nucleoside diminishing over time.

HPLC standard calibration of single nucleosides. In order to assess the utilization of HPLC and column in the quantitative analysis, standard calibration of the nucleosides was carried out. Standard solutions of each nucleoside at varying concentrations were injected into the HPLC and the area under peak determined. The detected peak area (uV*sec) was plotted versus the amount injected (pmole) and fitted to a linear equation (**Fig. S9**). Each point is an average of three measurements with error bars indicated (in most cases, the error bars are smaller than the symbol depicted).

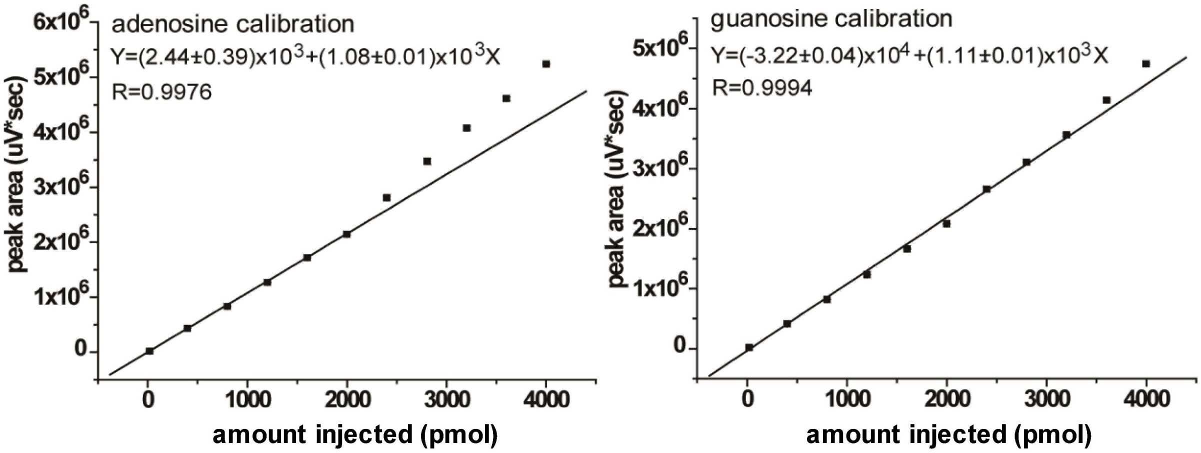

Figure S9. HPLC calibration curves for adenosine and guanosine are shown.

Characterization of AAPt-nucleoside adducts. Mass analysis of the fractions obtained from HPLC C18 separation of the AlaPt-Ado and OrnPt-Ado adducts was carried out using FT-ICR MS equipped with an ESI source. These measurements reveal that AlaPt-$Ado_{N1/N3}$ and AlaPt-Ado_{N7} are isomers with the same mass, charge, and isotopic distribution, corresponding to a monofunctional adduct, $[Pt(Ado)(Ala)(Cl)+H]^+$ (**Fig. S10**). The mass profiles of OrnPt-$Ado_{N1/N3}$ and OrnPt-Ado_{N7} also reveal that they are isomers with the same mass, charge, and isotopic distribution, corresponding to a monofunctional adduct, $[Pt(Ado)(Orn)(Cl)]^+$ (**Fig. S11**).

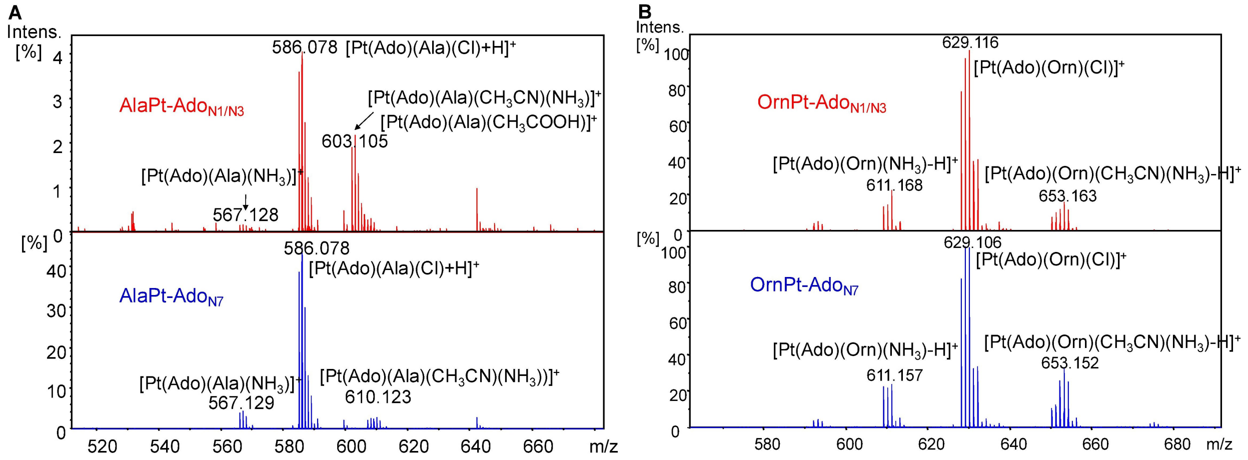

Figure S10. Mass analysis of the AAPt-Nuo fractions. (**a**) Mass analysis of AlaPt-Ado fractions (AlaPt-Ado$_{N1/N3}$ in red and AlaPt-Ado$_{N7}$ in blue), in which a monofunctional adduct species, $[Pt(Ado)(Ala)(Cl)+H]^+$, is observed in both fractions. (**b**) Mass analysis of OrnPt-Ado fractions (OrnPt-Ado$_{N1/N3}$ in red and OrnPt-Ado$_{N7}$ in blue), in which a monofunctional adduct species, $[Pt(Ado)(Orn)(Cl)]^+$ is observed in both fractions. Other adducts are also formed by the loss or addition of neutral molecules to the monofunctional adducts.

NMR characterization of Pt-Ado adducts. Two-dimensional (2D) heteronuclear single quantum correlation (HSQC) spectroscopy was employed to assign the peaks for the H8 and H2 protons of OrnPt-Ado$_{N1/N3}$ and OrnPt-Ado$_{N7}$ (**Fig. S11**). ^{1}H-NMR spectroscopy was then employed to determine the specific platination sites in the adducts by comparison with the chemical shifts of the H2 and H8 protons of Ado (**Fig. S12**). In the ^{1}H-NMR spectrum of OrnPt-Ado$_{N1/N3}$, the major peak corresponding to H8 (closed triangle) shows a small chemical shift change (< 0.1 ppm), whereas the other major peak corresponding to H2 (closed circle) exhibits a 0.5 ppm downfield shift, which suggests that the metal center is most likely coordinated to the N1 (or N3) position of Ado [6]. For the set of minor peaks corresponding to H2 (open circle) and H8 (open triangle), signals were shifted downfield by about 0.5 ppm suggesting a doubly platinated Ado. Therefore, the fraction of OrnPt-Ado$_{N1/N3}$ is a mixture of products consisting of OrnPt-Ado$_{N1/N3}$ monofunctional adduct and doubly platinated Ado, $[(OrnPt)_2(Ado)]$, with coordination at both the N1/N3 and N7 positions.

For OrnPt-Ado$_{N7}$, both peaks for H8 (triangles, open and closed for major products) are shifted downfield by approximately 0.5 ppm, while the corresponding changes in the H2 chemical shifts are only 0.1 ppm. The stronger downfield shift observed for the two H8 signals suggests that platination occurs on the neighboring N7 for both products. The dominant signal set (closed symbols) is assigned as the monofunctional OrnPt-Ado$_{N7}$ and the other set (open symbols) is assigned as a bifunctional adduct [OrnPt(Ado)$_2$] with coordination to both N7 positions (also confirmed by mass)

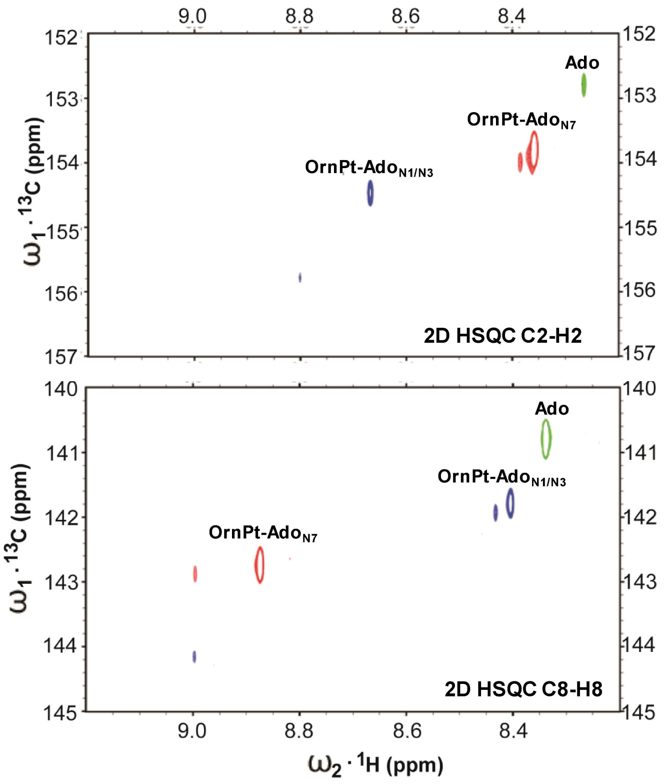

Figure S11. 2D heteronuclear single quantum correlation (HSQC) of OrnPt-Ado$_{N7}$ (top) and OrnPt-Ado$_{N1/N3}$ (bottom). In reference to the signal for Ado C2-H2 (top) [7], the major signal for OrnPt-Ado$_{N1/N3}$ exhibits platination at the N1 (or N3) position. On the other hand, in reference to the Ado C8-H8 (bottom) [7], the major peak for OrnPt-Ado$_{N7}$ exhibits platination at the N7 position. The minor signals observed are for doubly platinated or bifunctional adducts that elute with the major peaks during HPLC separation of the adducts.

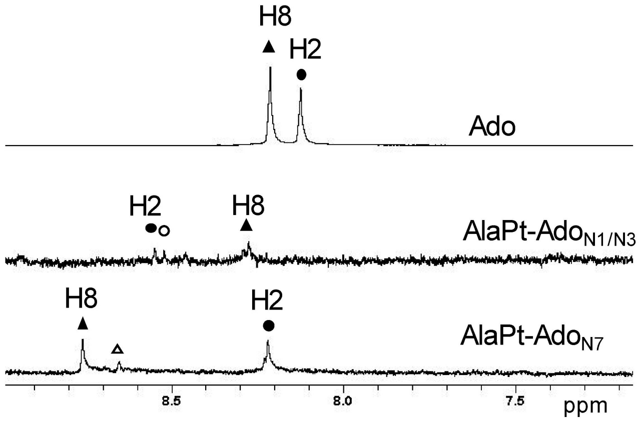

Figure S12. Aromatic region of 1D ^{1}H-NMR spectra of the compounds from two HPLC fractions. Ado (top), AlaPt-Ado$_{N1/N3}$ (middle) and AlaPt-Ado$_{N7}$ (bottom); proton signals assigned as H8 and H2 are noted with triangles and circles, respectively. The open circles are assigned as protons from other AlaPt-Ado species that have not been identified by more advanced NMR methods.

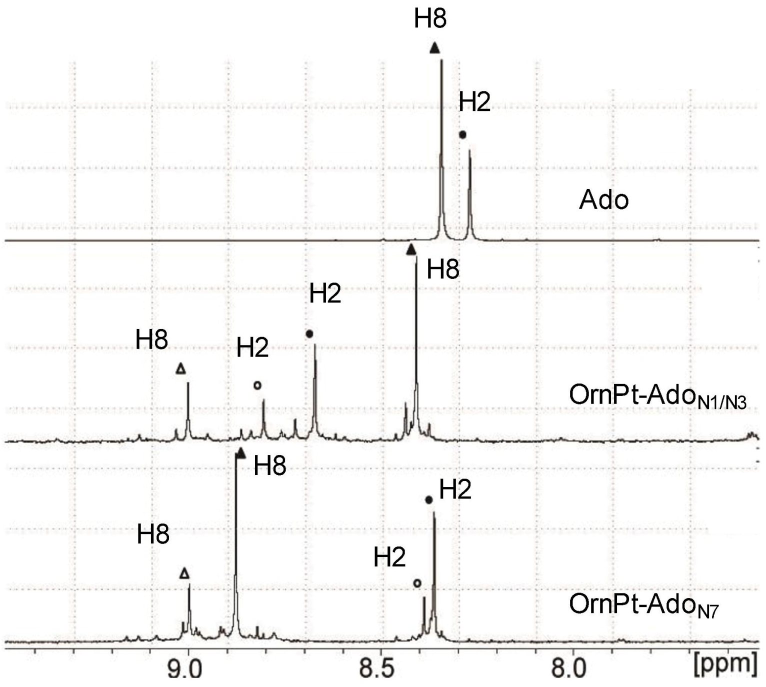

Figure S13. Aromatic region of ^{1}H-NMR spectra of the compounds from two HPLC fractions. Ado (top), OrnPt-Ado$_{N1/N3}$ (middle) and OrnPt-Ado$_{N7}$ (bottom); proton signals for H8 and H2 are noted with triangles and circles, respectively. The open circles and triangles are assigned as protons from doubly platinated and bifuctional species that have also been confirmed by mass analysis.

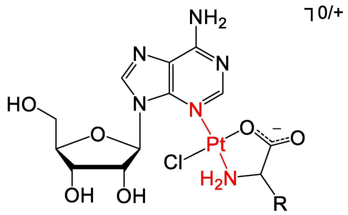

trans-Ado-N3
α-NH_2 is *trans* to N3

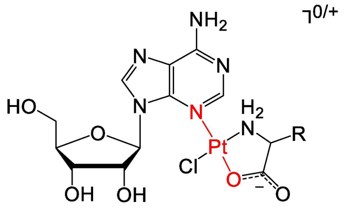

cis-Ado-N3
α-NH_2 is *cis* to N3

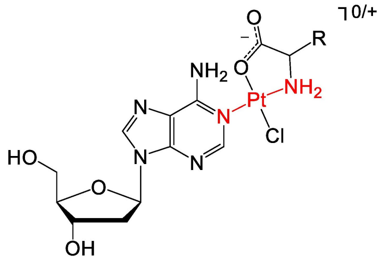

trans-dAdo-N1
α-NH_2 is *trans* to N1

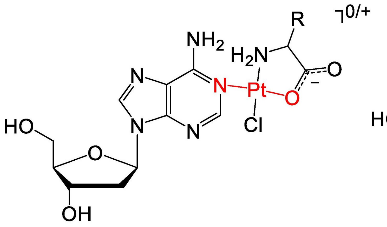

cis-dAdo-N1
α-NH_2 is *cis* to N1

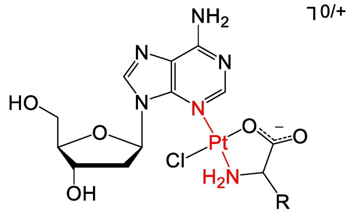

trans-dAdo-N3
α-NH_2 is *trans* to N3

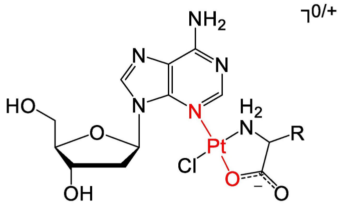

cis-dAdo-N3
α-NH_2 is *cis* to N3

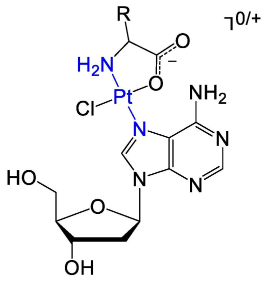

trans-dAdo-N7 adduct
α-NH_2 is *trans* to N7

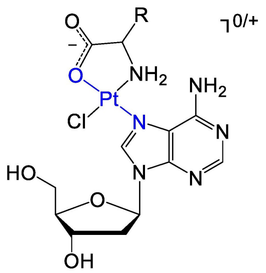

cis-dAdo-N7 adduct
α-NH_2 is *cis* to N7

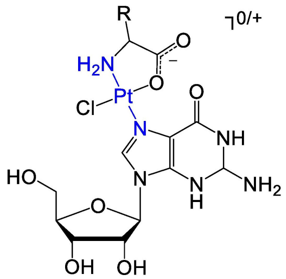

trans-Guo-N7 adduct
α-NH_2 is *trans* to N7

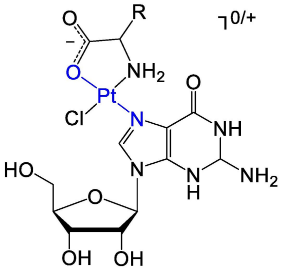

cis-Guo-N7 adduct
α-NH_2 is *cis* to N7

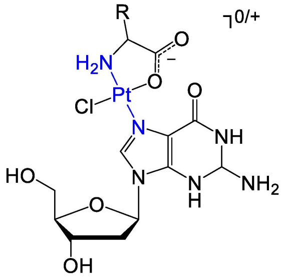

trans-dGuo-N7 adduct
α-NH_2 is *trans* to N7

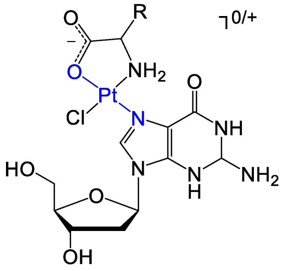

cis-dGuo-N7 adduct
α-NH_2 is *cis* to N7

Figure S14. Possible isomers and orientations of AAPt-purine adducts.

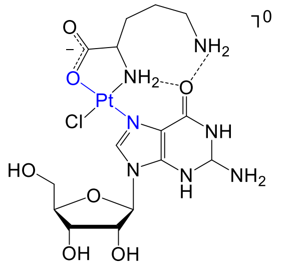

Figure S15. OrnPt-Guo$_{N7}$ with two stabilizing hydrogen bonds.

References

1. Still BM, Kumar PGA, Aldrich-Wright JR, Price WS (2007) Chem Soc Rev 36:665-686
2. Watabe M, Kai M, Goto K, Ohmuro H, Furukawa S, Chikaraishi N, Takayama T, Koike Y (2003) J Inorg Biochem 97:240-248
3. Dalla Via L, Gia O, Magno SM, Dolmella A, Marton D, Di Noto V (2006) Inorganica Chim Acta 359:4197-4206
4. Corbett JF (1972) J Chem Educ 49:663
5. Lee KW, Martin Jr DS (1976) Inorganica Chim Acta 17:105-110
6. Eastman A (1982) Biochemistry 21:6732-6736
7. Ulrich EL, Akutsu H, Doreleijers JF, Harano Y, Ioannidis YE, Lin J, Livny M, Mading S, Maziuk D, Miller Z, Nakatani E, Schulte CF, Tolmie DE, Kent Wenger R, Yao H, Markley JL (2008) Nucleic Acids Res 36:D402-D408
